# A variant of the autophagic receptor NDP52 counteracts phospho-TAU accumulation and emerges as a protective factor for Alzheimer’s disease

**DOI:** 10.1038/s41419-025-07611-2

**Published:** 2025-04-15

**Authors:** Anna Mattioni, Claudia Carsetti, Krenare Bruqi, Valerio Caputo, Valentina Cianfanelli, Maria Giulia Bacalini, Mariella Casa, Carlo Gabelli, Emiliano Giardina, Gianluca Cestra, Flavie Strappazzon

**Affiliations:** 1 IRCCS Fondazione Santa Lucia, Rome, Italy; 2 Institute of Molecular Biology and Pathology (IBPM), National Research Council (CNR), c/o University of Rome Sapienza, Rome, Italy; 3 Department of Biology and Biotechnology, University of Rome Sapienza, Rome, Italy; 4 Univ Lyon, Univ Lyon 1, CNRS, INSERM, Physiopathologie et Génétique du Neurone et du muscle, UMR5261, U1315, Institut Neuromyogène, Lyon, France; 5 Department of Clinical Medicine, Life, Health & Environmental Sciences-MESVA, University of Aquila, Aquila, Italy; 6 Department of Science, University “ROMA TRE”, Rome, Italy; 7 Department of Woman and Child Health and Public Health, Gynecologic Oncology Unit, Fondazione Policlinico Universitario A. Gemelli IRCCS, Rome, Italy; 8 IRCCS Istituto delle Scienze Neurologiche di Bologna, Bologna, Italy; 9 Department of Medicine, Clinical Center for the Aging Brain, University of Padua, Padova, Italy; 10 Department of Biomedicine and Prevention, Tor Vergata University, Rome, Italy

**Keywords:** Autophagy, Macroautophagy

## Abstract

Selective elimination of early pathological TAU species may be a promising therapeutic strategy to reduce the accumulation of TAU, which contributes to neurodegeneration and is a hallmark of Alzheimer’s disease (AD). Pathological hyper-phosphorylated TAU can be degraded through selective autophagy, and NDP52/CALCOCO2 is one of the autophagy receptors involved in this process. In 2021, we discovered a variant of NDP52, called NDP52^GE^ (rs550510), that is more efficient at promoting autophagy. We here anticipate that this variant could be a powerful factor that could eliminate pathological forms of TAU better than its WT form (NDP52^WT^). Indeed, we provide evidence that in in vitro systems and in a *Drosophila melanogaster* model of TAU-induced AD, the NDP52^GE^ variant is much more effective than the NDP52^WT^ in reducing the accumulation of pathological forms of TAU through the autophagic process and rescues typical neurodegenerative phenotypes induced by hTAU toxicity. Mechanistically, we showed that NDP52^WT^ and NDP52^GE^ bind pTAU with comparable efficiency, but that NDP52^GE^ binds the autophagic machinery (LC3C and LC3B) more efficiently than NDP52^WT^ does, which could explain its greater efficiency in removing pTAU. Finally, by performing a genetic analysis of a cohort of 435 AD patients, we defined the NDP52^GE^ variant as a protective factor for AD. Overall, our work highlights the variant NDP52^GE^ as a resilience factor in AD that shows a robust effectiveness in driving pathological TAU degradation.

## Introduction

Alzheimer’s disease (AD) is the most prevalent progressive neurological disorder affecting aged people worldwide [1, 2]. In the brain, amyloid-β (Aβ) plaques and TAU neurofibrillary tangles pathologically characterize AD and are associated with the loss of synapses and neurons, which results in dementia [3]. To date, no effective disease-modifying therapies are available, indicating a deeper understanding of the molecular mechanisms leading to neuronal death and the identification of new potential therapeutic targets [4, 5]. Accumulating evidence suggests that TAU pathology, which consists of TAU mislocalization, oligomerization, changes in solubility, and aggregation in filaments and neurofibrillary tangles, is better correlated with the degree of dementia than Aβ deposition is [6–8]. Furthermore, the early phases of TAU pathology, triggered mainly by the hyperphosphorylation of TAU, appear to correlate better with neuronal toxicity than the highly ordered filaments typical of the final phases of the disease [9–11]. The clearance of these early pathological TAU species, particularly hyperphosphorylated TAU, could therefore be a promising strategy for therapeutic interventions.

NDP52 (Nuclear Dot Protein 52, also known as CALCOCO2) is an autophagic receptor that has been shown to facilitate pathological phospho-TAU (pTAU) clearance via selective autophagy [12]. Mechanistically, as other autophagic receptors, NDP52 physically links specific cargoes committed to lysosomal degradation, with the autophagy core machinery responsible for the formation of the cup-shaped membrane called the phagophore. This initial step triggers the signaling cascade that allows cargo enwrapment by forming a double-membrane vesicle—the autophagosome—which then fuses with lysosomes for content degradation [13]. The interaction between the cLIR (noncanonical LC3-interacting region) motif of NDP52 and LC3C, an ATG8 family member anchored to the nascent phagophore, ensures the efficient engulfment of the cargo selected by NDP52 in the autophagosome. In addition, the binding of a distinct LIR motif of NDP52 with other ATG8 family members (LC3A/B and GABARAPs, but not LC3C), and the interaction with the motor protein MYOSIN VI, allow NDP52 to promote the autophagosome maturation, ensuring efficient cargo degradation [13, 14]. In mouse and rat cortical neurons, the silencing or overexpression of NDP52 resulted in increased or reduced levels of pathological pTAU, respectively. Moreover, in both the AD mouse brain and the AD human cerebral cortex, NDP52 interacts with pathological pTAU, and the SKICH (skeletal muscle and kidney-enriched inositol phosphatase (SKIP) carboxyl homology) domain of NDP52 binds TAU in vitro [12, 15]. Taken together, these data strongly suggest that NDP52 prevents TAU aggregation by clearing pathological pTAU, thereby slowing the progression of AD.

In 2021, we characterized a variant of NDP52, hereafter referred to as NDP52^GE^ (c.491G>A, rs550510, p.G140E), in which the G140E substitution enhances the ability of NDP52 to interact with LC3C. Indeed, this amino acid position is very close to the cLIR motif, and the presence of a glutamic acid residue (instead of a glycine) appears to induce a conformational change that favors the interaction with LC3C. We also showed that, compared with its wild-type form (NDP52^WT^), NDP52^GE^ promotes greater degradation of damaged mitochondria, a well-known cargo of NDP52 [16].

Here, we explored the hypothesis that NDP52^GE^, by recruiting more of the autophagic machinery, could promote greater autophagic degradation of pathological pTAU, countering its accumulation better than NDP52^WT^ and thus delaying the onset and/or progression of AD. Indeed, we demonstrated that this variant counteracts the accumulation of pathological TAU better than NDP52^WT^ does in two in vitro systems (SH-SY5Y P301S TAU inducible cell line and SH-SY5Y treated with okadaic acid (OkA)). Furthermore, we showed in vivo, in a *Drosophila melanogaster* model of TAU-mediated AD, that NDP52^GE^ partially rescued different pathological phenotypes and promoted the degradation of pathological forms of TAU. Finally, *via* genetic analysis, we showed that NDP52^GE^ is a protective factor in AD.

Overall, our work points to NDP52^GE^ as a novel, promising target for the development of innovative therapeutic interventions (i.e., genetic manipulation) in AD.

## Materials and methods

### Cell lines, transfection and reagents

SH-SY5Y cells came from an in-house stock and were maintained in DMEM-F12 Glutamax (Gibco, 31331-028), 10% Fetal bovine Serum (FBS) (Corning, 35-015-CV). Human SH-SY5Y P301S-TAU inducible cell line was a kind gift from Luc Buée [17] and was maintained in DMEM complemented with glutamax, non-essential amino acids, P/S, and 10% of FBS. To induce P301S-TAU expression, cells are maintained in medium with 1 µg/ml tetracycline (TET). Cells were maintained at 37 °C in a humidified atmosphere containing 5% CO_2_ and were trypsinized using 0.05% Trypsin-EDTA (Gibco, 25300-054). Transient DNA transfections were performed using Lipofectamine 2000 according to the supplier’s instructions (Invitrogen, 11668019). To induce hyper-phosphorylation of TAU, cells were treated with OkA (Santa Cruz Biotechnology, SC-3513). According to supplier’s instructions, OkA was dissolved at 100 μM in dimethyl sulphoxide (DMSO) (Sigma-Aldrich, D5879), and used at the final concentration of 100 nM for 1 h, 1.5 h, or 2 h. An equal amount of DMSO used in the OkA dilution was added to the control cells. To inhibit the autophagic process human SH-SY5Y P301S-TAU inducible cells were treated with 3-Methyladenine (3-MA) for 8 h. To block the autophagosome–lysosome fusion cells were treated with NH_4_Cl (Sigma-Aldrich/MERCK, Germany, 09718) at 20 mM for 24 h.

### Plasmids

HA-LC3C, FLAG-NDP52^WT^, and FLAG-NDP52^GE^ vectors were previously described [16]. DNA of hNDP52^WT^ or hNDP52^GE^ was subcloned as EcoRI/BamHI fragments into pEGFP-C1 vector for mammalian expression or subcloned into pUAST-attB vector, using EcoRI/BglII restriction sites, to generate *Drosophila* transgenic strains. p3xFLAG-CMV10-NDP52^WT^ or p3xFLAG-CMV10-NDP52^GE^ were used to generate the V136S and ΔLIR (from 203-DYWE-206 to 203-AAAA-206) mutant constructs by using the QuickChange Multi Site-Directed Mutagenesis kit (Agilent Technologies, 200515) according to the manufacturer’s instructions. The sequences used for mutagenesis are as follows:

FLAG-NDP52^V136S^:5′-GGAAGACATCCTGGTTTCTACCACTCAGGGAGAGGTG -3′;

FLAG-NDP52^GE_V136S^:5′-GGAAGACATCCTGGTTTCTACCACTCAGGAAGAGGTG -3′;

FLAG-NDP52^ΔLIR^ and FLAG-NDP52^GE_ ΔLIR^:

5′- GAAAGAACAGAAGGCCGCTGCGGCGACAGAGCTGCTTCAACTG -3′.

The constructs coding for GFP-GABARAP, GFP-GABARAPL1, and GFP-GABARAPL2 were kindly provided by Dr. P. Grumati (TIGEM, Naples, Italy. The constructs coding for HA-LC3A and HA-LC3B were kindly provided by C. Behrends (Ludwig-Maximilians-Universität (LMU) München, Germany). All constructs were systematically verified by DNA sequencing (BioFab Research, Rome, Italy).

### Western blotting analysis

Subconfluent cultures of SH-SY5Y cells were lysed in ice-cold lysis buffer (10 mM Tris-HCl pH 7.4, 150 mM NaCl, 1 mM EGTA, 0.25% Na-DOC, 0.1% SDS, 1% NP-40) supplemented with 1 mM NaF, 1 mM Na3VO4, 1 mM phenylmethyl sulphonyl fluoride (PMSF) and protease inhibitors (Roche Diagnostic, 11836153001). Samples were kept on ice for 30 min, then lysates were clarified by centrifugation at 13,000 rpm (=15700rcf) for 10 min at 4 °C. The concentration of proteins in the supernatants was determined using Bradford assay (Bio-Rad, 5000006). Supernatants were mixed with 4 × Laemmli sample buffer, boiled, resolved by SDS–PAGE and wet transferred overnight to PVDF (Millipore, Immobilon-P IPVH00010). Membranes were blocked with 5% non-fat dry milk in Tris-HCl-buffered saline (TBS) and incubated with the indicated primary and secondary antibodies diluted in 1%milk-TBS1x-0.025% tween-20 and then detected with Clarity Western ECL Substrate (Millipore, Immobilon, WBKLS0500). Densitometry analyses of western blots were performed using the ImageJ software. Antibodies used were as follow: mouse anti-FLAG M2 antibody (Sigma-Aldrich F1804), rabbit anti-FLAG (Sigma-Aldrich F7425), rabbit anti-HA (Sigma-Aldrich, H6908); rabbit anti-β-ACTIN (Sigma Aldrich, A2066); mouse anti-TAU (TAU46) (Santa Cruz Biotechnology, sc-32274); rabbit anti-pTAU Ser396 (Invitrogen, 44-752G); mouse anti-pTAU Ser202,Thr205 (AT8) (Invitrogen, MN1020); mouse anti-pTAU Thr212,Ser214 (AT100) (Invitrogen, MN1060); mouse anti-VINCULIN (Santa Cruz Biotechnology, sc-73614 clone 7F9); rabbit anti-NDP52 (Cell Signaling, 60732); mouse anti-NDP52 (Novus Biologicals, NBP2-03246, clone OTI4H5); rabbit anti-GABARAP+GABARAPL1+GABARAPL2, used to recognize Drosophila Atg8 (dAtg8) (Abcam; ab109364); rabbit anti-GIOTTO (was a gift from GL Cestra; https://www.antibodyregistry.org/AB_2892585); goat anti-mouse IgG (H+L)-HRP conjugated (Bio-Rad, 1706516); goat anti-rabbit IgG (H+L)-HRP conjugated (Bio-Rad, 1706515).

### Co-immunoprecipitation (Co-IP)

Subconfluent cultures of SH-SY5Y cells were lysed in ice-cold lysis buffer (50 mM Tris-HCl pH 7.4, 150 mM NaCl, Na-DOC 0.25%, Triton 1% NP-40 0.5%, glicerolo 10%) supplemented with 1 mM NaF, 1 mM Na3VO4, 1 mM PMSF and protease inhibitors (Roche Diagnostic, 11836153001). Samples were kept on ice for 30 min, then the lysates were clarified by centrifugation at 13,000 rpm (=15700rcf) for 10 min at 4 °C. Five hundred micrograms of total proteins from each sample were incubated with mouse anti-HA (Sigma-Aldrich, H3663) or mouse anti-FLAG M2 antibodies (Sigma-Aldrich F1804) on a rotational shaker for 2 h at 4 °C, and then incubated with Protein A-Agarose beads (Roche 11134515001) on a rotational shaker at 4 °C for 1 h. Samples were washed with three changes of Washing Buffer (50 mM Tris-HCl pH 7.4, 150 mM NaCl, NP-40 0.5%) supplemented with 1 mM NaF, 1 mM Na3VO4, 1 mM PMSF, and protease inhibitors (Roche Diagnostic, 11836153001). For each change, samples were centrifuged at 2400 rpm (=500rcf) for 3 min at 4 °C, and the supernatants carefully removed. After the final wash, the pellets were mixed with 4 × Laemmli sample buffer, and western blotting analysis was performed as described above.

### Immunofluorescence

**Fig. 1:**
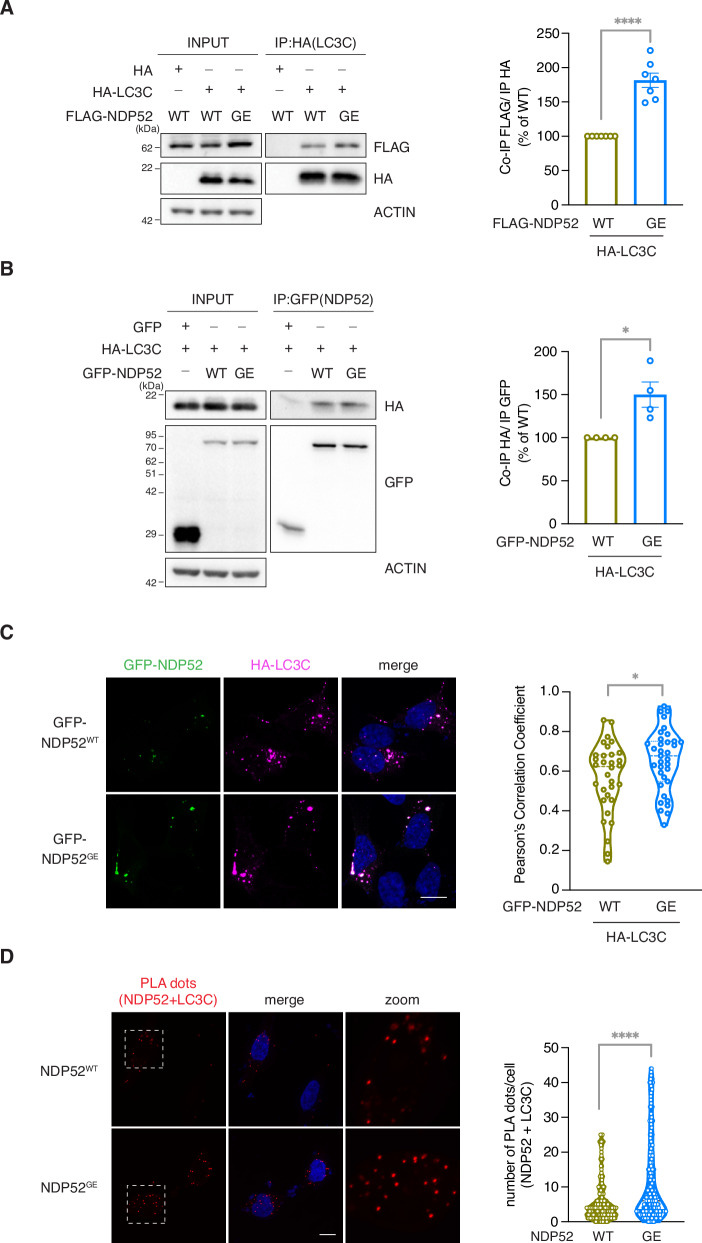
NDP52^GE^ binds LC3C more efficiently than NDP52^WT^ in a human neuroblastoma cell line. **A** Lysates of SH-SY5Y cells expressing the indicated FLAG- and HA-tagged proteins was immunoprecipitated with anti-HA beads. Samples were analyzed by Western blot using the indicated antibodies. The graph reports the amount of the indicated FLAG-NDP52 protein coprecipitated by the corresponding HA-LC3C protein. Data were expressed as percentage variation over FLAG-NDP52^WT^. Images and data are representative of seven independent experiments. **B** Lysates of SH-SY5Y cells expressing the indicated GFP- and HA-tagged proteins were immunoprecipitated with anti-GFP beads. Samples were analyzed by Western blot using the indicated antibodies. The graph reports the amount of HA-LC3C coprecipitated by the corresponding GFP-NDP52 protein. Data were expressed as percentage variation over GFP-NDP52^WT^. Images and data are representative of four independent experiments. **C** Representative confocal images of SH-SY5Y cells expressing the indicated GFP- and HA-tagged proteins, fixed and stained with anti-HA (magenta staining) antibodies and with DAPI (blue staining in the merge panels) to detect nuclei. Colocalization of GFP-NDP52 and HA-LC3C was quantified by measuring the Pearson’s Correlation Coefficient. The results are reported in the graph. Images are representative of three independent experiments, and at least 30 cells for each condition were analyzed. Scale bar: 10 μm. In (**B**, **C**) data are presented as means ± SEM. *p < 0,05; ***p = <0.001 (two-tailed unpaired *t*-test).

SH-SY5Y cells, grown on coverslips, were washed in warm PBS1x (GIBCO, BE17-512F), and fixed with warm 4% paraformaldehyde in PBS1x for 10 min at 37 °C. After permeabilization with 0.4% Triton X-100 (Sigma-Aldrich, X-100) in PBS 1x for 5 min, cells were incubated at 4 °C for 24 h in PBS1x, 2% normal goat serum (Sigma-Aldrich, G9023) and the primary antibodies. Cells were then washed with PBS1x and incubated for 1 h in PBS1x, 2% normal goat serum, and the labeled secondary antibodies. After washes with PBS1x, cells were stained with 1 μg/ml of 4,6-diamidino-2-phenylindole (DAPI) to detect the nuclei, and coverslips were mounted with Fluoromount Mounting Media (Sigma-Aldrich, F4680). Antibodies used were as follow: mouse anti-HA (Sigma-Aldrich, H3663), rabbit anti-NDP52 (Cell Signaling, 60732), mouse anti-TAU (TAU46) (Santa Cruz Biotechnology, sc-32274), rabbit anti-FLAG (Sigma-Aldrich F7425), mouse anti-FLAG M2 antibody (Sigma-Aldrich F1804), anti-mouse Alexa Fluor^TM^ 488 (Thermo Fisher Scientific, A11017), anti-mouse Alexa Fluor^TM^ 555 (Thermo Fisher Scientific A21425), anti-rabbit Alexa Fluor^TM^ 488 (Thermo Fisher Scientific, A11070) and anti-rabbit Alexa Fluor^TM^ 555 (Thermo Fisher Scientific, A21430). Figure 1C: samples were analyzed with a Zeiss LSM 800 microscope equipped with 63x oil-immersion objectives. Images were acquired using ZEN system (ZEISS Germany). All acquisitions were performed in non-saturated serial Z-stacks, taking care to cover the entire cell volume. Colocalization analysis was performed by measuring, for each transfected individual cell, the Pearson’s correlation coefficient through the JACoP plugin [18] of the IMAGEJ software [19]. Figure 5C, D: samples were acquired using Nikon Eclipse Ti microscope equipped with 100x oil-immersion objective. TAU intensity was measured using ImageJ/Fiji software [19].

### Lambda phosphatase (λPP) treatment

SH-SY5Y treated with OkA as described above were lysed in ice-cold lysis buffer (50 mM Tris-HCl pH 7.4, 150 mM NaCl, Na-DOC 0.25%, Triton 1% NP-40 0.5%, glycerol 10%) supplemented with 1 mM PMSF, 2 mM DTT, and protease inhibitors (Roche Diagnostic, Germany, 11836153001). Samples were kept 15 min on ice, then the lysates were clarified by centrifugation at 13,000 rpm (=15700rcf) for 10 min at 4 °C. Supernatants were collected, and 10xλPP buffer and 10xMnCl_2_ were added. Each sample was split into two fractions, one was left untreated (as control) while in the other 2 μl of λPP enzyme (400 U/μl; Santa Cruz sc-200312A) were added. After incubation at 30 °C for 1 h, samples were mixed with 4 × Laemmli sample buffer and Western blotting analysis was performed as described above.

### In situ proximity ligation assay (PLA)

PLA was performed on cells grown on coverslips, fixed in 4% paraformaldehyde, and permeabilized with 0.1% Triton X-100-PBS1x for 5 min. The Duolink in situ PLA kit (Sigma-Aldrich DUO92101) was used according to manufacturer’s instructions. The amplification time was 140 min for all tested interactions. Primary antibodies (codes specified in the Western Blotting analysis section) were used in pairs to detect the interactions, or alone as negative control. Figure 3E: SH-SY5Y cells were transfected with plasmids coding FLAG-NDP52^WT^ or FLAG-NDP52^GE^ and after 8 h of expression treated with DMSO (basal condition) or with 100 nM OkA for 1.5 h at 37 °C. To detect the interactions between NDP52 constructs and TAU, rabbit anti-FLAG and mouse anti-TAU primary antibodies were used. Cell nuclei were stained and slides mounted on coverslips using the manufacturer’s mounting medium containing DAPI. Samples were analyzed using the Nikon Eclipse Ti2-E confocal spinning disk inverted microscope. Serial Z stacks were acquired, taking care to cover the entire cell volume. PLA dots were counted using Cell Profiler software [20].

### *Drosophila melanogaster* strains and procedures

Drosophila stocks were maintained on Drosophila standard medium (Nutri-fly Genesee Scientific, El Cajon, CA, USA) at 25 °C unless otherwise specified. Fly line carrying UAS-hTAU^2N4R^ (51362 (w[1118]: P{w[+mC]=UAS-Tau.wt}1.13); RRID:BDSC_51362) was obtained from the Bloomington Stock Center (https://bdsc.indiana.edu). Fly lines carrying hNDP52^WT^ or hNDP52^GE^ were generated by phiC31-mediated transgenesis by BestGene Inc (https://www.thebestgene.com/HomePage.do). pUAST-attB constructs were inserted on the same genomic landing site on the third chromosome. Eye or panneuronal transgene expression was achieved using the eyeless-GAL4 or elav-GAL4 driver, respectively. For the measure of the eye area, adult flies of both sexes were analyzed. Pictures of both fly eyes were taken at the higher magnification using the stereomicroscope (ZEISS Semi 508, 50X) (Zeiss) equipped with the Axiocam camera 105 and exploiting ZEISS ZEN software (Blue edition 3.1). Eye area was then measured using NHI ImageJ FIJI freeware software (National Institute of Health, Bethesda, USA) [19]. Measurements were shown using a violin plot in Graphpad Prism 8. More than 150 eyes per genotype were analyzed. For the climbing assay flies were crossed and maintained at 29 °C. Negative geotaxis assays were performed according to the standard protocols [21]. Since the viability of male flies expressing hTAU was strongly reduced in these conditions, we assessed locomotory activity of female adult flies. Female flies were collected and placed in new vials for 24 h to eliminate the effect of CO_2_ anesthesia. The flies were then transferred in empty vials for 1 h before to be placed in the climbing assay holder. The climbing ability has been scored by tapping flies to the bottom of the vial and scoring how many flies have reached the target line (4 cm) after 10 s. Flies were tested in batches of 10–15 and three trials were performed on each analysis. More than 40 flies per genotype were employed in this assay. Data were reported using a Box and whiskers (min to max) plot in Graphpad Prism 8. Lifespan was assessed maintaining groups of 5 virgin flies of same sex in each vial (more of 100 female and 40 males per genotype) and keeping them at 29 °C. Flies were transferred on fresh food daily and death events were recorded until the last survivor died. Data were plotted on prism GraphPad survival tables. Elapsed days were entered on the X axis and the percentage of survival on the Y axis. Mantel-Cox analysis was used to test the statistical significance of pairs of survival curves. For western blotting analysis 10 heads of adult flies from both sexes and for each genotype were homogenized. The lysis buffer and the western blotting procedures were performed as described in the previous Western Blotting analysis paragraph.

### Statistical analysis

The number of independent biological replicates and the sample size (not pre-determined) were indicated in the respective figure legends. No blinded analysis was performed. All statistical tests, indicated in the respective figure legends, were performed and represented graphically using GraphPad Prism 8 software (San Diego, California, USA).

### Genetic association case-control analysis

#### Study cohorts

The study cohort overall involved 434 patients affected by sporadic AD and 1000 healthy subjects as control group. Blood samples from 189 AD patients and 1000 control subjects already stored in the Genomic Medicine Laboratory of Santa Lucia Foundation IRCCS were analyzed. AD patients were characterized by a F:M ratio = 66:34 and a mean age ± s.d. = 74.9 ± 7.55. Briefly, these patients were recruited from 2010 to 2021 at the Outpatient Memory Clinic of the Laboratory of Neuropsychiatry of IRCCS Santa Lucia Foundation, Rome, Italy. The diagnosis of sporadic AD’s dementia was based on medical history and neurological examination, including brain imaging and instrumental tests, overall fulfilling the clinical criteria of the National Institute on Aging and the Alzheimer’s Association [22]. A more detailed description can be found in [23]. These patients were enrolled for a previous study approved by the Ethical Committee (CE/PROG.650 approved on 01/03/2018) of IRCCS Santa Lucia Foundation Hospital of Rome and in accordance with the Declaration of Helsinki. A written informed consent of the study was obtained for all patients and control subjects. In addition, blood samples from 245 AD untreated patients were collected from 245 untreated patients admitted to the memory clinic of CRIC in Padua between April 2001 and March 2018. Diagnosis of late onset non-familial AD was made, according to internationally established criteria. The diagnosis was obtained at the end of the diagnostic protocol by clinical, laboratory, and neuropsychological assessment, all subjects underwent CT or MR neuroimaging, and 84/245 were further characterized after PET scanning or CSF biomarkers evaluation. All peripheral blood samples were collected after obtaining the written informed consent of the study participants, in accordance with the Helsinki Declaration. The study protocol “Analisi delle Basi Biologiche della Malattia di Alzheimer eredofamiliare e delle forme sporadiche tardive” (3950/AO/2016) was approved by the local ethic committee of the recruitment center on October 06th, 2016.

#### Genomic DNA extraction

Genomic DNA was extracted from 200–400 μL of whole blood by means of MagPurix Blood DNA Extraction Kit using MagPurix Automatic Extraction System (Resnova) according to the manufacturer’s instructions. The obtained DNA samples were assessed for quantity and purity byT by DeNovix Spectrophotometer (Resnova), obtaining a concentration range of 50–150 ng/μL and A260/230 and A260/280 ratios included between 1.7 and 1.9.

#### Genotyping analysis

The extracted DNA was employed for the genotyping analysis for the rs550510 (G/A) variant of interest using a predesigned TaqMan assay on QuantStudio® 5 Real-Time PCR System (Applied Biosystems). Each real-time PCR run was performed using a negative control and three positive samples that were previously tested by direct sequencing (BigDye Terminator v3.1) and run on ABI3130xl (Applied Biosystems).

#### Statistical analyses

The genotyping results related to all patients and controls have been tested for evaluating the association and the effect of the rs550510 (G/A) variant as described in [16]. Briefly, as reference group, 1000 healthy subjects were employed. The Hardy–Weinberg equilibrium was confirmed in both cases’ and controls’ cohorts. The obtained data were then evaluated by calculating a *P* value (*p*) through a 2 × 2 (allele association) and 2 × 3 (genotype association) contingency tables. The statistical associations were considered significant for *p* < 0.05 with a 95% confidence interval. ORs were calculated for evaluating the strength of the association.

## Results

### In the human SH-SY5Y neuroblastoma cell line, the NDP52^GE^ variant binds LC3C more efficiently than NDP52^WT^

**Fig. 2:**
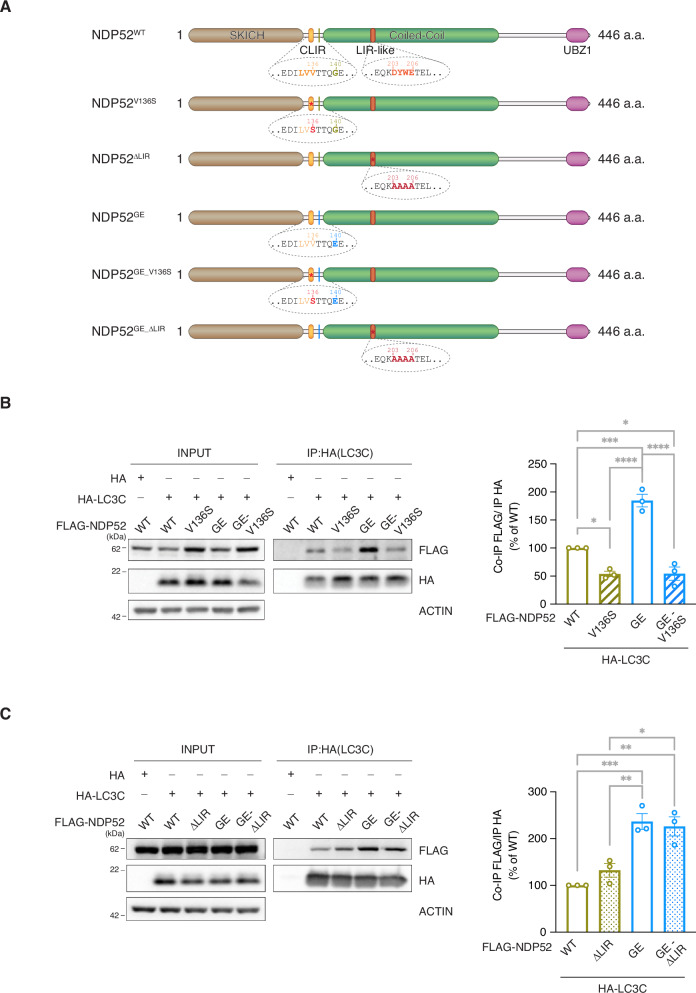
The increased binding affinity of NDP52^GE^ towards LC3C strictly depends on the cLIR motif. **A** Schematic representation of the human NDP52^WT^ protein, the human variant NDP52^GE^, and the NDP52 mutants used in this study. The G140E aminoacidic substitution is highlighted, and the position relative to cLIR motif as well as the LIR-like motif are shown in the expanded dotted area. SKICH skeletal muscle and kidney-enriched inositol phosphatase carboxyl homology, cLIR non-canonical LC3-interacting region, LIR LC3-interacting region, UBZ ubiquitin-binding zinc finger. Lysates of SH-SY5Y cells expressing the indicated FLAG- and HA-tagged proteins were immunoprecipitated with anti-HA (**B**) or anti-FLAG (**C**) beads. Samples were analyzed by Western blot using the indicated antibodies. The graphs report the amount of the indicated FLAG-NDP52 protein coprecipitated by the corresponding HA-LC3C protein. Data were expressed as percentage variation over FLAG-NDP52^WT^. Images and data are representative of three independent experiments. ****p-value < 0.0001; ***p-value < 0.001; **p-value < 0.01: *p-value < 0.05 (Ordinary One-way ANOVA, Turkey’s multiple comparisons test).

In 2021, we discovered that NDP52^GE^ binds more LC3C than its WT form does. To verify whether this was also the case in neuronal-like cells, we compared the binding ability of the NDP52^GE^ variant and NDP52^WT^ to LC3C in the human neuroblastoma SH-SY5Y cell line (Fig. 1). Moreover, we also compared, for the first time, their ability to bind to the other members of the ATG8 family. To this end, we performed co-immunoprecipitation assays from cells overexpressing each ATG8 family member with NDP52^WT^ or NDP52^GE^. The results revealed that both NDP52 proteins strongly interact with LC3C (Fig. 1), while their binding to LC3B and LC3A was weak (Supplementary Fig. S1B) and those to GABARAPs were even lower (Supplementary Fig. S1A). Moreover, NDP52^GE^ binding to GABARAPs showed an efficiency comparable to that of NDP52^WT^ (Supplementary Fig. S1A), whereas NDP52^GE^ binding to LC3A tended to increase compared with that of NDP52^WT^. However, this difference was not statistically significant. In contrast, NDP52^GE^ binds LC3B (Supplementary Fig. S1B) and LC3C (Fig. 1A) more efficiently than does NDP52^WT^. As the interaction with LC3C is the strongest among the ATG8 family members, we next focused on this binding. We confirmed the ability of NDP52^GE^ to bind LC3C more efficiently than NDP52^WT^ by performing co-immunoprecipitation in the opposite direction (Fig. 1B) and evaluating the colocalization between LC3C and NDP52^WT^ or NDP52^GE^ by immunofluorescence coupled with confocal microscopy analysis (Fig. 1C). Finally, we assessed whether the enhanced interaction between NDP52^GE^ and LC3C required the cLIR motif alone or the contribution of other NDP52 motifs. For each NDP52 protein, we generated two mutants in which either the cLIR motif (V136S) or the LIR-like motif (203-AAAA-206, ΔLIR) was functionally inactivated. These are the motifs of NDP52 involved in the binding to LC3C and to the other ATG8 family members respectively [14, 16]. Interestingly, they are both close to the G140E substitution (the NDP52 constructs are schematically represented in Fig. 2A). We performed Co-IP experiments to test the ability of these mutants to bind LC3C. The results revealed that both the NDP52^WT^ and NDP52^GE^ proteins mutated in the cLIR motif showed a drastic reduction in the ability to co-precipitate LC3C (Fig. 2B). In contrast, NDP52 proteins whose LIR-like motif was mutated, maintained the ability to co-precipitate LC3C and, similar to NDP52^GE^, the NDP52^GE_ΔLIR^ mutant bound to LC3C more efficiently than the NDP52^WT_ΔLIR^ mutant did (Fig. 2C). Taken together, these data confirmed that the NDP52^GE^ variant interacts with LC3C more efficiently than NDP52^WT^ in neuronal-like cells and that the increased binding affinity is strictly dependent on the cLIR motif of NDP52.

### NDP52^GE^ delays the accumulation of hyperphosphorylated forms of TAU in OkA-treated neuronal-like cells better than NDP52^WT^

**Fig. 3:**
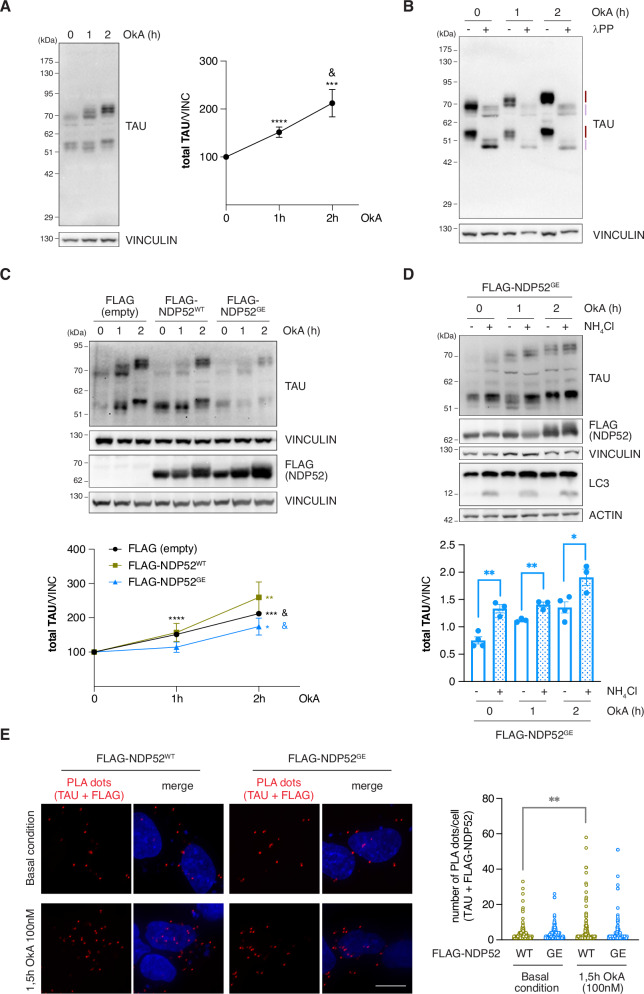
NDP52^GE^ reduces pTAU levels better than NDP52^WT^, in a human neuroblastoma cell line. **A** Western blot (WB) on extracts of SH-SY5Y cells treated with 100 nM OkA for the indicated times. TAU bands were measured, summed, and normalized to the corresponding signal of VINCULIN. Results are expressed as percentage variation of the levels at time 0. Images and data are representative of eight independent experiments. **B** Lysates of SH-SY5Y cells treated as in (**A**) were split and incubated with (+) or without (−) Lambda Protein Phosphatase (λPP). Images are representative of three independent experiments. Lilac lines = non-phosphorylated TAU; purple lines = phosphorylated TAU. **C** SH-SY5Y cells expressing the indicated FLAG-tagged proteins were treated as in (**A**). Signals from TAU antibody were measured on WB and normalized as in (**A**). Results are expressed as percentage variation of the levels measured at time 0 for each FLAG-tagged protein expressing group. Images and data are representative of eight independent experiments and are reported as means ± SEM. * indicates comparison to time 0 of each FLAG-tagged protein expressing group; & indicates comparison between 1 h and 2 h of each group (two tailed unpaired *t*-test) **D** SH-SY5Y cells expressing FLAG-tagged NDP52^GE^ were pre-treated with NH_4_Cl (20 mM, 24 h) or not, and then were treated with 100 nM OkA as indicated. Signals from TAU antibody were measured on WB and normalized as in (**A**). Images and data are representative of three independent experiments and are reported as means ± SEM. At each OkA time, NH_4_Cl non-treated vs NH_4_Cl treated results were tested for statistical significance (two-tailed unpaired *t*-test). **E** Representative confocal images of in situ PLA performed in SH-SY5Y overexpressing FLAG-NDP52^WT^ or FLAG-NDP52^GE^ treated with DMSO or with 100 nM OkA for 1.5 h at 37 °C. Dots were counted using CellProfiler software and results were shown in the violin plot graphs. At least 100 cells for each condition were analyzed. (Ordinary One-way ANOVA, Turkey’s multiple comparisons test). Scale bar: 10 μm.

Since we confirmed that NDP52^GE^ binds LC3C more efficiently than NDP52^WT^ does, we expected that this variant would be able to recruit autophagosomes better, resulting in a faster degradation of its cargoes. Considering the previous demonstration that NDP52^WT^ facilitates the clearance of pathological hyperphosphorylated TAU (pTAU) by autophagy [12], we wondered whether NDP52^GE^ would promote faster degradation and, in turn, a slower accumulation of pTAU. To verify this hypothesis, we again used the neuroblastoma SH-SY5Y cell line since it expresses all six human TAU (hTAU) isoforms and shows a TAU phosphorylation state similar to that of the human brain [24]. Moreover, we used OkA, a potent pharmacological inhibitor of multiple protein phosphatases, to induce TAU hyperphosphorylation and simulate the early events of TAU-specific pathology [25–28]. As shown in Fig. 3A, under basal conditions (Fig. 3A, lane 0), the anti-TAU signal resulted in several bands with different electrophoretic mobilities grouped into two regions: one at ~50–60 kDa and the other at ~70 kDa. This distribution is consistent with the molecular weights of the six TAU isoforms, which range from 48 kDa to 67 kDa. The treatment of SH-SY5Y cells with 100 nM OkA for 1 h and 2 h induced a progressive increase in the intensity and a shift in the electrophoretic mobility of the signals positive for TAU (Fig. 3A, compare lane 0 with lane 1 and lane 2). The incubation of OkA-treated lysates with lambda phosphatase (λPP) enzyme, which removes phosphate groups from proteins, reversed the shift in the electrophoretic mobility of the TAU-positive signals (Fig. 3B), confirming that OkA treatment effectively induced the formation of hyperphosphorylated TAU. In this model, we next tested the effects of NDP52^WT^ and NDP52^GE^ overexpression on the levels of hyperphosphorylated TAU. As shown in Fig. 3C, TAU accumulated more slowly in cells overexpressing NDP52 than in the control, reaching a statistically significant increase only after 2 h of OkA treatment. In addition, NDP52^GE^ appears to be more powerful than NDP52^WT^ in slowing pTAU accumulation. However, when cells overexpressing NDP52^GE^ were treated with ammonium chloride (NH_4_Cl) to inhibit lysosomal degradation of the autophagic cargoes, pTAU accumulated much more (Fig. 3D), indicating that NDP52^GE^ mediates TAU clearance through the autophagy process.

At this stage, we cannot exclude the possibility that NDP52^GE^ is also able to bind TAU more efficiently than is NDP52^WT^. To address this point, we examined the interaction of NDP52 proteins with TAU or the hyperphosphorylated forms of TAU induced by OkA treatment. We performed in situ PLA experiments on SH-SY5Y cells overexpressing NDP52^WT^ or NDP52^GE^ under basal conditions or upon OkA treatment (Fig. 3E). Under basal conditions, the results revealed PLA dots for both NDP52^WT^ and NDP52^GE^, indicating that interactions with TAU also occur at a non-pathological level. The number of PLA dots did not significantly differ between cells overexpressing NDP52^GE^ or NDP52^WT^, suggesting that NDP52 proteins bind TAU with comparable efficiency. Similarly, upon OkA treatment, PLA dots were observed and, again, the number of PLA dots was not significantly different in cells overexpressing NDP52^GE^ or NDP52^WT^. These findings suggest that the hyperphosphorylated forms of TAU are bound by both NDP52 proteins with similar efficiencies. Compared with that in the basal condition, the number of PLA dots increased upon OkA treatment. However, this increase was statistically significant in cells overexpressing NDP52^WT^ but not in those overexpressing NDP52^GE^. This could most likely be explained by the fact that NDP52^GE^ degrades the hyperphosphorylated forms of TAU more efficiently than NDP52^WT^ does (Fig. 3C) so that the NDP52^GE^/phospho-TAU complexes are likely removed faster than those with NDP52^WT^. Although further experiments will be needed to confirm this latter point, our results clearly indicate that NDP52^WT^ and NDP52^GE^ do not differ in their binding affinity to TAU or its hyperphosphorylated forms.

### NDP52^GE^ promotes the autophagic degradation of pathological P301S-TAU more efficiently than NDP52^WT^

**Fig. 4:**
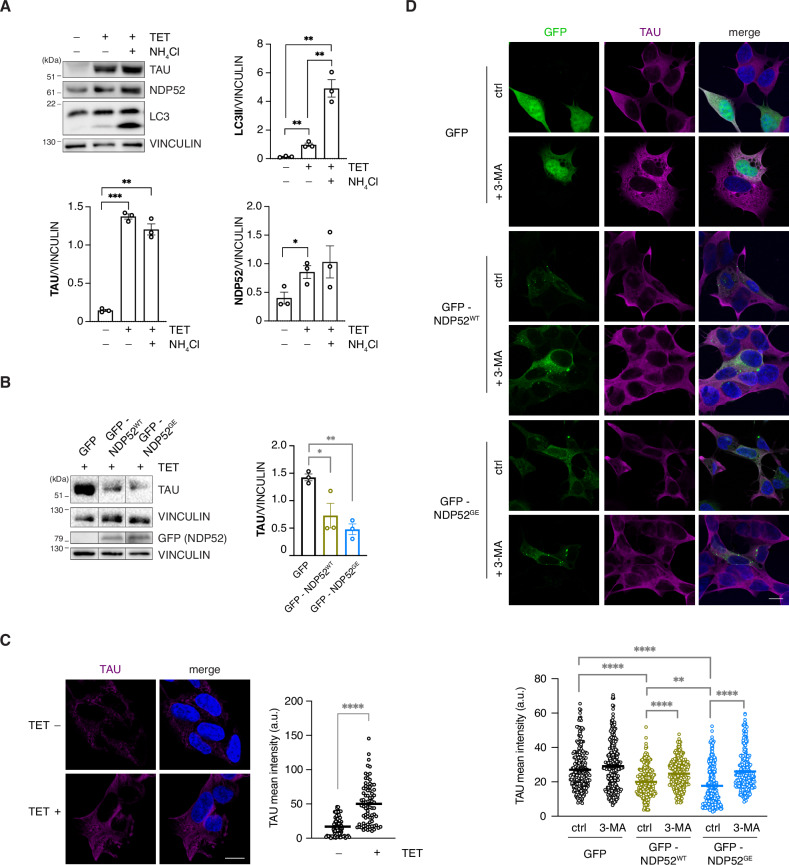
NDP52^GE^ degrades P301S-TAU via autophagy and is more efficient than NDP52^WT^. **A** WB analysis of human SH-SY5Y P301S-TAU inducible cells non-treated (TET −) or treated (TET +) with tetracycline for 24 h to induce P301S-TAU expression. To block the autophagosome–lysosome fusion, cells were treated with NH_4_Cl (20 mM, 24 h). Images are representative of three independent experiments. Signals from LC3II, TAU, and NDP52 antibodies were measured and normalized to the corresponding signal of VINCULIN. Data are reported as means ± SEM. (two-tailed unpaired *t*-test). **B** WB analysis of human SH-SY5Y P301S-TAU inducible cells overexpressing GFP, GFP-NDP52^WT^ or GFP-NDP52^GE^ and treated with tetracycline to induce P301S-TAU expression. After 16 h, the medium was substituted with a TET-free medium for 8 h in order to evaluate and compare TAU degradation rate. Data are reported as means ± SEM. (two-tailed unpaired *t*-test). **C** Representative confocal images of human SH-SY5Y P301S-TAU inducible cells non-treated (TET −) or treated (TET +) with tetracycline for 24 h to induce P301S-TAU expression, fixed and stained with anti-TAU (magenta staining) antibody and with DAPI (blue staining in the merge panels) to detect nuclei. Intensity of anti-TAU signal was measured, and results are reported in the graph. Images are representative of three independent experiments and more than 70 cells for each condition were analyzed. (two-tailed unpaired *t*-test). Scale bar: 10 μm. **D** Representative confocal images of human SH-SY5Y P301S-TAU inducible cells overexpressing GFP-NDP52^WT^ or GFP-NDP52^GE^ and treated as in (**B**). Cells were left untreated (ctrl) or were treated with the autophagic inhibitor 3-Methyladenine (+3-MA) for 8 h. Cells were fixed and stained with anti-TAU antibody and with DAPI to detect nuclei. Intensity of anti-TAU signal was measured, and results are reported in the graph. Images are representative of three independent experiments and more than 200 cells for each condition were analyzed. (two-tailed unpaired *t*-test).

To further investigate the involvement of NDP52 in the clearance of pathological TAU, we took advantage of a different in vitro system, the P301S mutant TAU inducible SH-SY5Y cell line [17]. First, we confirmed the induction of P301S-TAU expression upon TET treatment (Fig. 4A, C) and monitored the endogenous levels of LC3 and NDP52. Interestingly, both the LC3-II and the endogenous NDP52 protein levels increased upon TET treatment (Fig. 4A). Moreover, LC3-II levels strongly increased upon NH_4_Cl treatment, suggesting that the expression of P301S-TAU increases autophagosome formation and activates the autophagy pathway, possibly involving NDP52.

Our previous results revealed that NDP52^WT^ and NDP52^GE^ slowed the accumulation of early pathological pTAU. However, in the OkA-treated system we cannot distinguish whether TAU expression was reduced and/or TAU turnover was increased. To assess this point, GFP (as a control) or GFP-NDP52^WT^ or GFP-NDP52^GE^ constructs were overexpressed, and P301S-TAU was induced by TET treatment in inducible SH-SY5Y cells. After 16 h, the medium was replaced with TET-free medium to stop P301S-TAU expression, and 8 h later, the levels of TAU were measured *via* western blot analysis. As shown in Fig. 4B, the levels of P301S-TAU were significantly reduced in cells overexpressing NDP52^WT^ or NDP52^GE^. In addition, NDP52^GE^ tends to favor the degradation of pathological TAU more strongly than NDP52^WT^ does. To strengthen these data, we performed a similar experiment in which the levels of TAU were measured via an immunofluorescence assay coupled with confocal microscopy analysis. The results revealed that NDP52^WT^ overexpression significantly reduced the level of P301S-TAU compared with that in the control (Fig. 4D). Moreover, the NDP52^GE^ variant plays a more efficient role in mediating this process than does NDP52^WT^. This result suggests that NDP52 exerts its effect on TAU most likely by enhancing TAU degradation, rather than affecting TAU expression. To verify this hypothesis, cells were treated with 3-MA, a well-known autophagy inhibitor. In this case, both NDP52^WT^ and NDP52^GE^ failed to reduce the level of P301S-TAU (Fig. 4D), confirming that NDP52 proteins mediate TAU clearance through the autophagy process.

### Human NDP52 ameliorates TAU-mediated toxicity in an in vivo AD model of *Drosophila melanogaster*, and the variant NDP52^GE^ proves to be more powerful than NDP52^WT^

**Fig. 5:**
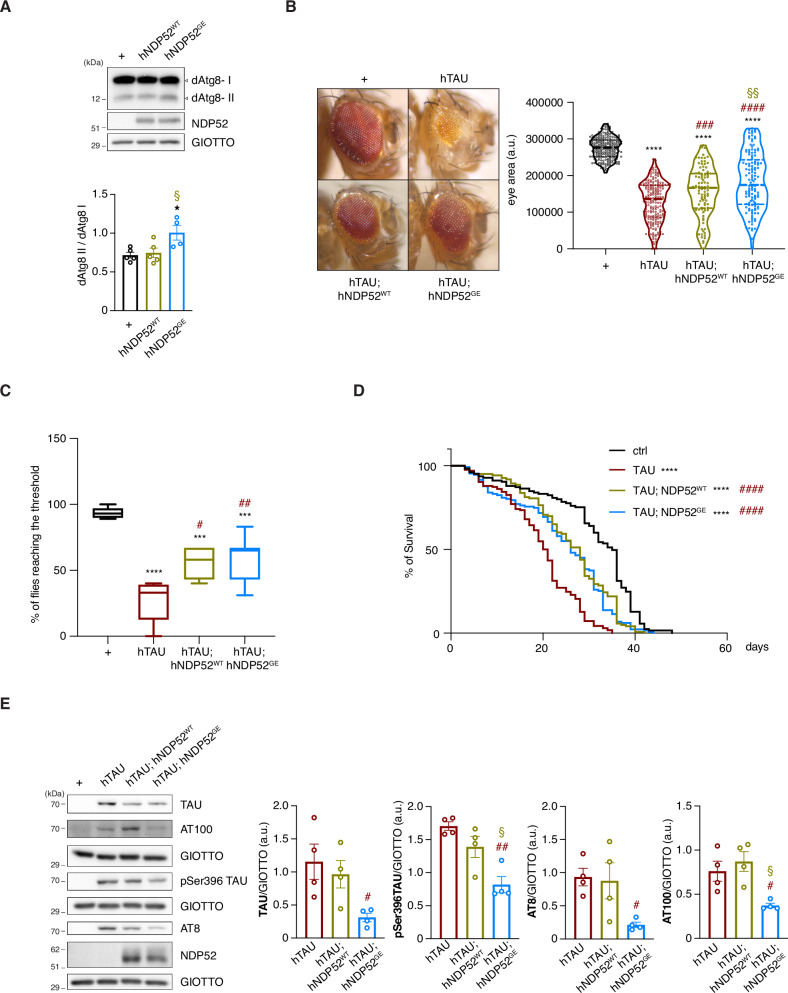
hNDP52^GE^ improves hTAU phenotypes in *Drosophila melanogaster* better than hNDP52^WT^. **A** Lysates from heads of flies expressing the indicated transgenes in the eye, at 29 °C, under control of GMR-GAL4 driver were analyzed by WB using the indicated antibodies. + refers to non-transgenic flies used as control. Images are representative of four independent experiments. The ratio between dAtg8II/dAtg8I was calculated and reported in the graph as means ± SEM. * indicates comparison of dAtg8II/dAtg8I between flies expressing hNDP52^GE^ and control; § indicates comparison of dAtg8II/dAtg8I between flies expressing hNDP52^GE^ and hNDP52^WT^ (Ordinary One-way ANOVA, Turkey’s multiple comparisons test). **B** Representative images of eye from flies expressing the indicated human (h) transgenes at 25 °C under control of eyeless-GAL4 driver. Violin plot below reports eye areas. Median and quartiles are shown as dotted lines. More than 150 eyes from both males and females flies were measured for each condition. * indicates comparison to control (+); # indicates comparison to hTAU; § indicates comparison to hTAU;hNDP52^WT^ (Ordinary One-way ANOVA, Turkey’s multiple comparisons test). **C** Box and whiskers (min to max) plot showing the percentage of flies reaching the threshold. Female flies expressed panneuronally (elav-GAL4) the indicated human transgenes at 29 °C. More than 40 flies per genotype were analyzed (Control *n* = 54 flies; hTAU *n* = 44; hTAU, NDP52^WT^ *n* = 53; hTAU, NDP52^GE^ *n* = 48 flies). * indicates comparison to control (+); # indicates comparison to hTAU (Ordinary One-way ANOVA, Turkey’s multiple comparisons test). **D** Survival curve showing lifespan of female flies expressing panneuronally (elav-GAL4) the indicated transgenes at 29 °C. * indicates comparison to control (+); # indicates comparison to hTAU (Log-rank (Mantel-Cox) test). **E** Lysates from heads of flies expressing panneuronally (elav-GAL4) the indicated transgenes at 29 °C were analyzed by WB using the indicated antibodies. Images are representative of four independent experiments. Signals from the indicated TAU antibodies were measured and normalized to the corresponding signal of GIOTTO. Results are expressed as means ± SEM. # indicates comparison to hTAU; § indicates comparison to hTAU;hNDP52^WT^ (Ordinary One-way ANOVA, Turkey’s multiple comparisons test).

Our results revealed that NDP52 reduces the accumulation of pathological TAU, with the variant NDP52^GE^ being stronger than NDP52^WT^. To assess whether these effects would ameliorate the neurodegeneration induced by the accumulation of pathological TAU, we used an in vivo system. In particular, we used a *Drosophila* model of TAU toxicity that expresses the longest hTAU isoform (2N4R, hereafter referred to as hTAU) under the UAS promoter and generated two transgenic fly lines expressing either human NDP52^WT^ or NDP52^GE^ cDNA, downstream of the UAS promoter. These constructs were inserted at the same genomic site to avoid any variability in expression due to the genomic environment. We used the UAS-GAL4 system [29] to specifically express the human transgenes in different fly tissues. First, we evaluated whether the expression of hNDP52^WT^ or hNDP52^GE^ affects the ratio between the lipidated and unlipidated forms of dAtg8 (dAtg8 II/dAtg8 I ratio). Interestingly, we discovered that the expression of hNDP52^GE^ in fly eyes (under the control of the GMR-GAL4 driver), increased the dAtg8 II/dAtg8 I *ratio*, compared with that in the control and NDP52^WT^ strains (Fig. 5A). This result suggests that NDP52 could interact with the *Drosophila* autophagic machinery, and that NDP52^GE^ may be able to favor the autophagosome formation better than NDP52^WT^ also in *Drosophila melanogaster*.

Subsequently, we evaluated the effect of co-expressing hTAU and hNDP52^WT^ or NDP52^GE^. The expression of hTAU in the early phases of eye development (under the control of the *eyeless*-GAL4 driver) resulted in retina degeneration linked to a significant reduction in the eye area (Fig. 5B, Ey> vs Ey>hTAU) [30]. This reduction is partially rescued by either NDP52^WT^ or NDP52^GE^. Interestingly, the rescue effect was more significant in flies expressing hNDP52^GE^ (Fig. 5B). In this context we also monitored the levels of TAU and pTAU (pSer396TAU, AT8 and AT100) proteins by western blot analysis. No signals were detected for AT100 and AT8 antibodies. Interestingly, the levels of pSer396TAU were significantly lower in the strains co-expressing hTAU with both human variants of NDP52 compared to the strain expressing only hTAU. Moreover, the level of hTAU protein was lower in the strain co-expressing hTAU and hNDP52^GE^ than in the strain expressing only hTAU (Supplementary Fig. S2A). These findings strongly suggest that NDP52^GE^ counteracts TAU accumulation more efficiently than does NDP52^WT^.

To further explore the beneficial effect of hNDP52 expression on hTAU-induced toxicity, we performed a locomotor assay using a panneuronal driver (*elav*-GAL4). Compared with control flies, hTAU-expressing flies presented a statistically significant reduction in locomotor ability (Fig. 5C). Flies expressing either NDP52^WT^ or NDP52^GE^ showed partial rescue of the climbing activity. Although not statistically significant, a slight improvement in the climbing performance was observed in flies expressing hNDP52^GE^ compared with those expressing hNDP52^WT^. In addition, as the neurodegeneration strongly affects fly viability, we performed a lifespan assay to compare hTAU toxicity in the different *Drosophila* strains. Compared with those of flies expressing only hTAU, the lifespans of both females (Fig. 5D) and males (Supplementary Fig. S2B) flies co-expressing hNDP52^WT^ or hNDP52^GE^ significantly increased.

Finally, we tested whether the co-expression of hNDP52^WT^ or hNDP52^GE^ affected the protein levels of TAU and pTAU species in fly neurons. Although the expression of NDP52^WT^ was not sufficient to produce a statistically significant reduction in the TAU and pTAU protein levels (Fig. 5E), the expression of NDP52^GE^ significantly reduced the levels of TAU and pTAU (pSer396TAU, AT8 and AT100) compared to controls. Moreover, compared with NDP52^WT^ flies NDP52^GE^ flies presented statistically significant reductions in pSer396TAU and AT100. Collectively, our data indicate that NDP52 mitigates hTAU-induced neurotoxicity in vivo. Moreover, they showed that NDP52^GE^ promotes better rescue of *Drosophila* phenotypes induced by hTAU expression. In addition, considering that the way in which NDP52 transgenic fly lines were generated ensures the same level of expression and that under all the tested conditions NDP52^GE^ protein levels were comparable to those of NDP52^WT^ (Supplementary Fig. S2A–C, D), our data strongly suggest that the beneficial effect of NDP52^GE^ does not result from an increase in protein stability but rather from its greater capacity to promote the autophagy-mediated elimination of TAU proteins.

### The variant NDP52 G140E is associated to and protective in AD

Collectively, our data indicate that the NDP52^GE^ variant strongly reduces the levels of pathological TAU both in vivo and in vitro. Since this variant is present in the human population, we next explored whether this variant could be a possible novel protective factor for AD patients. To this end, we performed a genetic association case-control analysis on a cohort of 434 AD patients and 1000 control subjects. The results, reported in Table 1, revealed that the frequency of the A allele (c.491G>A, rs550510, p.G140E), which codes for NDP52^GE^, was lower in the AD group (cases) than in the control group. The biostatistical analysis indicated that the association with the disease was statistically significant (*p* = 0.0042), and that the variant allele *A* was protective in AD (Odds Ratio, OR *A* = 0.72, 95%CI: 0.58–0.90) (Table 1). Similarly, the frequencies of the heterozygous genotype (GA) and the homozygous genotype for the variant allele (AA) were lower in the AD group than in the control group. Additionally, the biostatistical analysis confirmed that the associations with the disease are statistically significant (*p* = 0.017), with the homozygous genotype for the variant allele (AA) being protective in AD (OR AA = 0.52, 95% CI: 0.26–1.05). Taken together, these results indicate that the NDP52^GE^ variant is a protective factor in AD.

**Table 1:** NDP52 c.491G>A (rs550510, p.G140E) associates with a decreased susceptibility to AD^a^.

Gene	SNP	Allele	Allele frequencies (cases)	Allele frequencies (controls)	*p*	OR (95% CI)	
*NDP52*	rs550510 G/A	G	0.858	0.814	0.0042	G: 1.39 (1.11 – 1.73)	
		A	0.142	0.186		A: 0.72 (0.58 – 0.90)	
		**Genotype**	**Genotype frequencies (cases)**	**Genotype frequencies (controls)**			**OR (95% CI) GG vs AA; GA vs AA**
		GG	0.740	0.667	0.017	-	GG: 1.93 (0.95 – 3.90)
		GA	0.237	0.293		GA: 0.73 (0.56 – 0.95)	GA: 1.41 (0.68 – 2.91)
		AA	0.023	0.040		AA: 0.52 (0.26 – 1.05)	–

^a^Results of the biostatistical analysis performed on the genotyping results of 434 AD patients and 1000 healthy control subjects.

*SNP* single nucleotide polymorphism, *OR* odd ratio, *CI* confidence interval.

## Discussion

Autophagy appears to be the primary route of clearance of TAU in healthy neurons and its dysfunction leads to the accumulation of TAU oligomers and insoluble aggregates [31]. Growing evidence suggests that these species are the most toxic to neurons [9–11] and strongly correlate with the synaptic dysfunction underlying memory deficits in AD [32, 33]. In contrast, the induction of autophagy can significantly reduce the formation of these species and thus could offer protection against TAU pathogenicity [4, 31, 34–36]. Preserved autophagy, accompanied by significantly reduced TAU pathology and maintenance of cognitive integrity, has been observed in resilient individuals who have AD neuropathology but remain non-demented [37]. Interestingly, in the hippocampus of these resilient AD patients, the protein levels of the autophagic receptor NDP52 are increased and negatively correlate with the levels of TAU oligomers [37]. Moreover, NDP52 has been shown to target pathological hyperphosphorylated TAU to the autophagic degradative pathway, possibly contributing to the prevention of TAU aggregation by acting as an autophagy receptor that promotes TAU clearance [12].

**Fig. 6:**
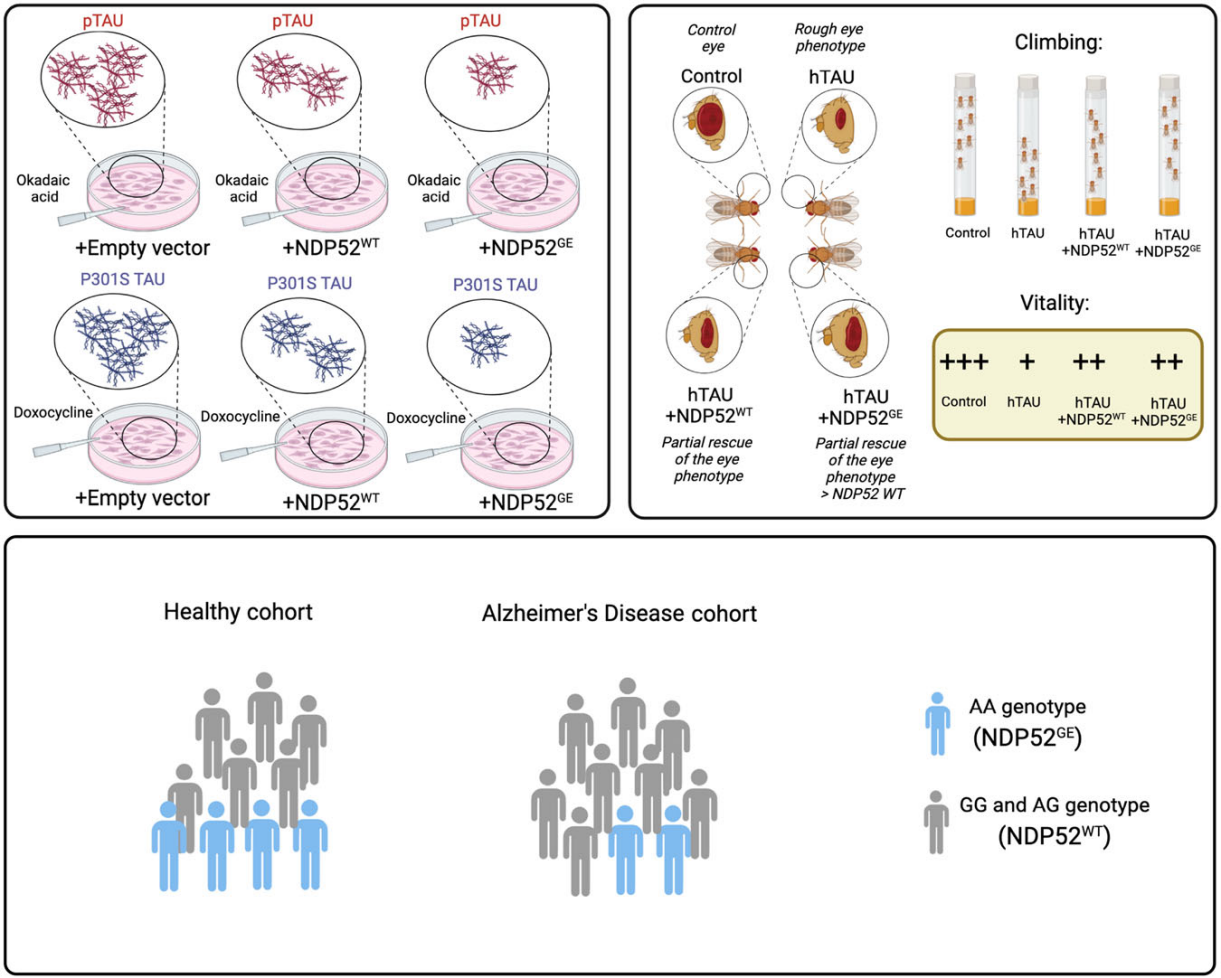
NDP52^GE^ is a protective factor in AD and counteracts pTAU accumulation. Illustration of the highlights of the work. By using two in vitro systems, we showed that the NDP52^GE^ variant counteracts the accumulation of pathological pTAU better than NDP52^WT^, through the autophagy process. Furthermore, we showed in vivo, in a *Drosophila melanogaster* model of TAU-mediated AD that the NDP52^GE^ variant partially rescues different pathological phenotypes induced by hTAU expression. Finally, using a genetic association case-control analysis, we showed that NDP52^GE^ is a protective factor in AD. Created with BioRender.com.

Here, we showed that NDP52^GE^, a variant of NDP52, is significantly associated with a decreased susceptibility to AD, resulting a protective factor for it. Consistent with the literature, we demonstrated that NDP52 directly binds hyperphosphorylated pathological forms of TAU and promotes their degradation through autophagy. Moreover, we showed in two different in vitro systems that NDP52^GE^ variant reduced the accumulation of pathological P301S-TAU and hyperphosphorylated forms of TAU better than NDP52^WT^. By taking advantage of a *Drosophila melanogaster* in vivo model of TAU-mediated AD, we demonstrated that NDP52 mitigates hTAU-induced neurotoxicity, and we confirmed a prominent effect of NDP52^GE^ compared with NDP52^WT^ (Fig. 6).

Mechanistically, we anticipated that the NDP52^GE^ variant was able to “clean” pathological hyper-phosphorylated TAU better than NDP52^WT^ through a stronger autophagy-mediated degradation. Indeed, our data indicate that NDP52^WT^ and NDP52^GE^ do not differ in their binding affinity for TAU or hyperphosphorylated TAU. In contrast, we confirmed that, through its cLIR motif, the NDP52^GE^ variant binds LC3C more efficiently than does NDP52^WT^. The cLIR-LC3C interaction is crucial for the efficient engulfment of cargoes into the autophagosomes [38], thus, the enhanced interaction with LC3C, induced by the presence of the E at position 140 [16], could promote more efficient autophagic clearance of cargo selected by NDP52, such as pathological hyperphosphorylated TAU proteins (similar to what has been previously observed for damaged mitochondria [16]). Interestingly, the residues of LC3C crucial for binding to the cLIR motif of NDP52, are highly conserved among Atg8 orthologs [38, 39]. In particular, the *Drosophila* Atg8a protein (dAtg8a), which is homologous to mammalian LC3, conserves all the residues responsible for the preferential binding of hLC3C to hNDP52, except for a single residue (Phe33 in hLC3C replaced by Tyr in dAtg8a) [38, 39]. Our findings that, also in *Drosophila melanogaster*, NDP52^GE^ clears pathologically phosphorylated TAU more efficiently than NDP52^WT^ might therefore support the hypothesis that NDP52^GE^/LC3C binding plays a pivotal role in promoting TAU degradation.

However, we cannot exclude the possibility that other mechanisms allow NDP52^GE^ to promote autophagy better than NDP52^WT^. While the NDP52/LC3C interaction allows cargo targeting to the nascent phagophore, binding to LC3A/B allows for autophagosome maturation, ensuring efficient cargo degradation [14]. Although the interaction with LC3C was the strongest among the ATG8 family members, we showed that NDP52^GE^ binds LC3B more efficiently than NDP52^WT^. Thus, the increased affinity for LC3B may also contribute to the ability of NDP52^GE^ to drive autophagy more efficiently than NDP52^WT^. In addition, it is known that NDP52 oligomerizes and, in the context of mitophagy, this facilitates the recruitment of the autophagic machinery around mitochondria for rapid degradation [40]. We cannot exclude the possibility that the NDP52^GE^ variant is able to oligomerize more efficiently than NDP52^WT^. This would promote stronger recruitment of the autophagic machinery around pathologically phosphorylated TAU, resulting in more rapid cargo degradation. Moreover, it has been shown that NDP52 restricts seeded TAU aggregation by diverting TAU seeds to autophagy, limiting the amplification and propagation of TAU inclusions throughout the brain [41]. In the future, testing whether NDP52^GE^ is more effective than NDP52^WT^ in these functions is important.

Ultimately, a recent paper by Santos et al. revealed that autophagy impacts on NDP52 nuclear localization and links NDP52 nuclear functions to gene transcription and DNA structure regulation [42]. It would be interesting to determine whether NDP52 affects TAU degradation also indirectly, by upregulating the transcription of factors involved in TAU clearance and whether NDP52^GE^ has greater nuclear localization and/or activity than NDP52^WT^.

Notably, NDP52 expression has been confirmed in both neurons and glial cells [15]. Given that microglia-driven neuroinflammation is a key factor that promotes TAU pathology development and spreading in AD [41, 43, 44], it would be interesting in the future to understand whether the beneficial role of NDP52^GE^ is limited to neurons or not, and whether it can be beneficial for neuroinflammation. It should be noted that we originally discovered this variant as a protective factor against Multiple Sclerosis (MS), an autoimmune disease of the central nervous system that is accompanied by neurodegeneration. In this context, we found that NDP52^GE^ was able to eliminate damaged mitochondria more efficiently than was NDP52^WT^, which could in turn reduce the inflammation associated with MS [16]. Mitochondrial dysfunctions have also been shown to be associated with AD [45]. In the future, it would be important to understand whether the beneficial effects of NDP52^GE^ in the context of AD are also related to the ability of NDP52^GE^ to eliminate damaged mitochondria more efficiently than NDP52^WT^.

Overall, our work highlights the variant NDP52^GE^ as a resilience factor in AD and highlights its robust effectiveness in slowing pathological TAU accumulation both in vitro and in vivo, suggesting great potential for therapeutic interventions in AD patients. Considering the current challenges in finding effective treatments to slow the progression of AD and improve patients‘ quality of life, this work may serve as a template for future explorations in the field of therapeutic chemistry and translational medicine.

## Supplementary information

Supplementary Figures

Uncropped WB

## Data Availability

All data generated during this study and supporting the present results are available from the corresponding author upon reasonable request.
