# Supplementary material for: A variant of the autophagic receptor NDP52 counteracts phospho-TAU accumulation and emerges as a protective factor for Alzheimer’s disease
Source: Cell Death Dis. 2025 Apr 15;16(1):300. doi: 10.1038/s41419-025-07611-2 (PMC12000434; doi:10.1038/s41419-025-07611-2)
Supplement: Supplementary file 1 — Supplementary Figures [file 41419_2025_7611_MOESM1_ESM.pdf]

A

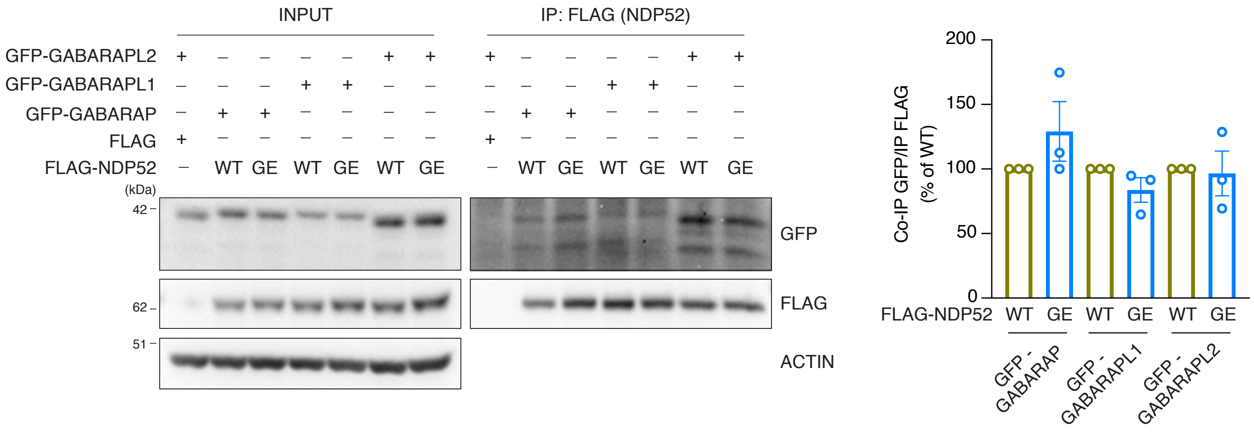

B

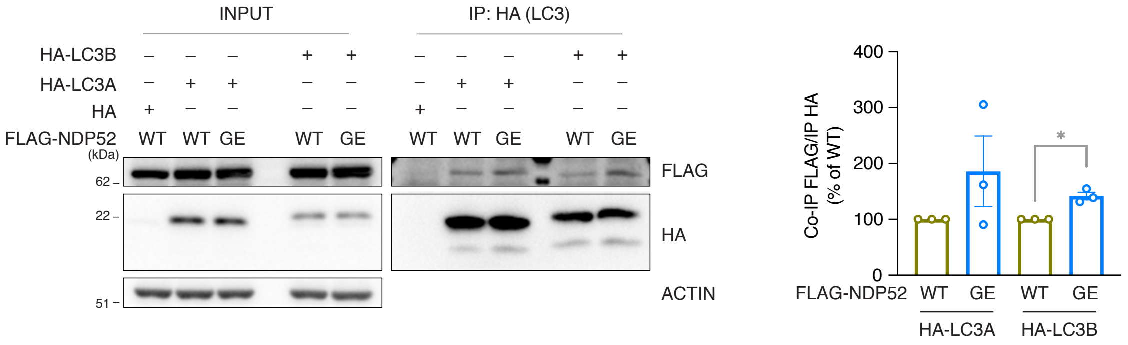

Supplementary Figure S1

Supplementary Figure S1. **$NDP52^{GE}$ binds LC3B more efficiently than $NDP52^{WT}$ in a human neuroblastoma cell line.** (**A**) Lysates of SH-SY5Y cells expressing the indicated GFP- and FLAG-tagged proteins were immunoprecipitated with anti-FLAG beads. Samples were analyzed by western blot using the indicated antibodies. The graph reports the amount of the indicated GFP-GABARAP protein coprecipitated by the corresponding FLAG-NDP52 protein. Data were expressed as percentage variation over FLAG-$NDP52^{WT}$. Images and data are representative of three independent experiments. Data are presented as means ± SEM (two tailed unpaired *t*-test: not statistical). (**B**) Lysates of SH-SY5Y cells expressing the indicated HA- and FLAG- tagged proteins were immunoprecipitated with anti-HA beads. Samples were analyzed by western blot using the indicated antibodies. The graph reports the amount of FLAG-NDP52 coprecipitated by the corresponding HA-LC3 protein. Data were expressed as percentage variation over FLAG-$NDP52^{WT}$. Images and data are representative of three independent experiments. Data are presented as means ± SEM. *$p<0,05$ (two tailed unpaired *t*-test).

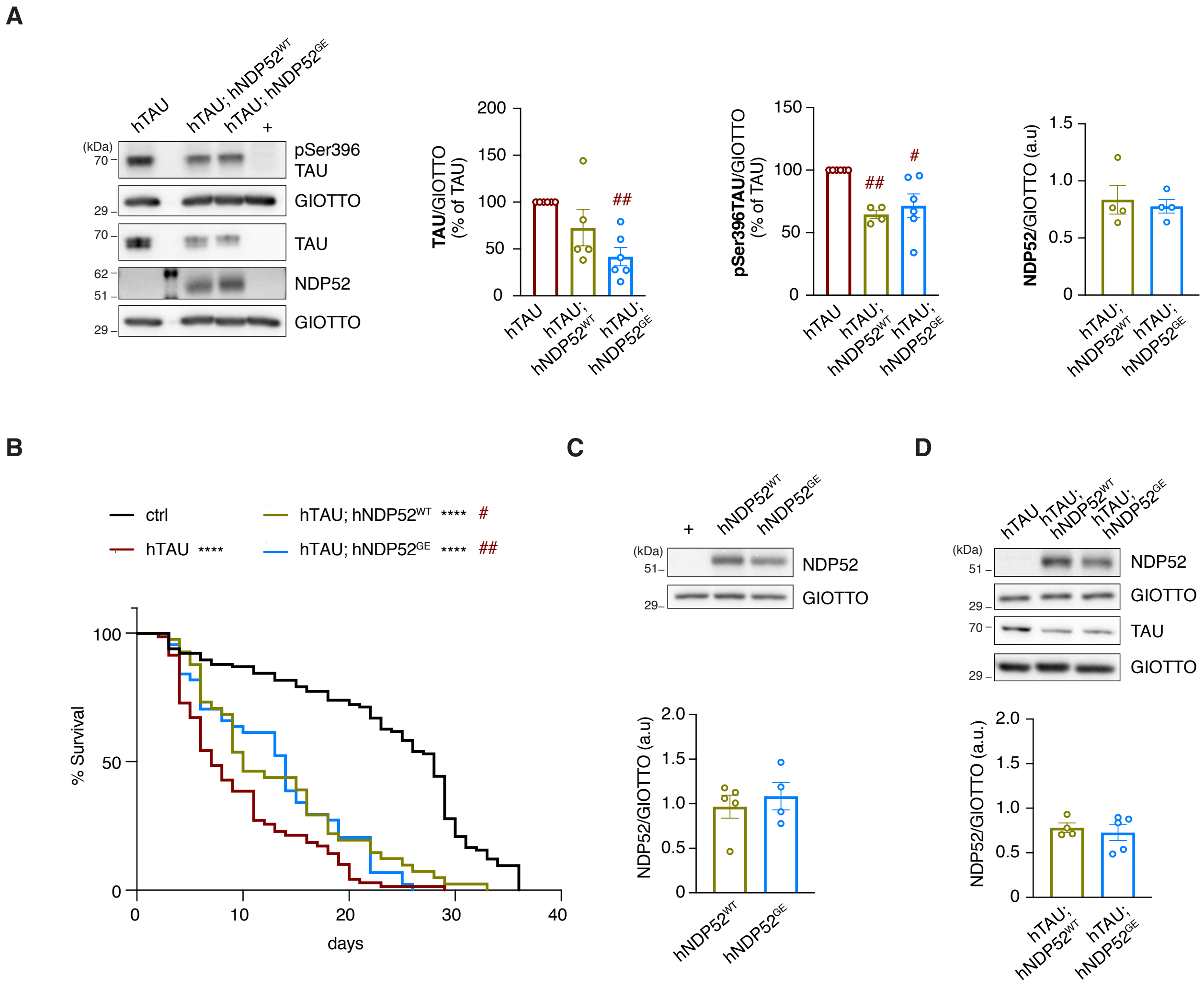

Supplementary Figure S2

Supplementary Figure S2. hNDP52 partially rescues hTAU phenotypes in *Drosophila melanogaster*. (**A**) Lysates from heads of flies expressing the indicated human (h) transgenes at 25°C under control of eyeless-GAL4 driver (Ey>) were analyzed by western blot using the indicated antibodies. + indicates non-transgenic flies used as control. Images are representative of 5 independent experiments. Signals from the indicated TAU and NDP52 antibodies were measured and normalized to the corresponding signal of GIOTTO, used as a loading control. Results, expressed as means ± SEM, are shown in the graphs. # indicates comparison to hTAU: # pvalue < 0,05, ## pvalue < 0,01 (Ordinary One-way ANOVA, Turkey's multiple comparisons test). (**B**) Survival curve showing lifespan of male flies expressing panneuronally (elav-GAL4, ELAV>) the indicated transgenes at 29°C. * indicates comparison to control (+): **** pvalue <0,0001; # indicates comparison to hTAU: # pvalue < 0,05; ## pvalue <0,01 (Log-rank (Mantel-Cox) test). (**C and D**) Lysates from heads of flies expressing the indicated transgenes under control of GMR-GAL4 driver (Ey>) (**C**) or panneuronally (elav-GAL4, ELAV>) (**D**) were analyzed by western blot using the indicated antibodies. The "+" sign refers to non-transgenic flies used as control. Images are representative of at least 4 independent experiments. Signals from NDP52 antibody were measured and normalized to the corresponding signal of GIOTTO, used as loading control. Results, expressed as means ± SEM, are shown in the graphs (two tailed unpaired *t*-test: not statistical).
