# Supplementary material for: A variant of the autophagic receptor NDP52 counteracts phospho-TAU accumulation and emerges as a protective factor for Alzheimer’s disease
Source: Cell Death Dis. 2025 Apr 15;16(1):300. doi: 10.1038/s41419-025-07611-2 (PMC12000434; doi:10.1038/s41419-025-07611-2)

FIGURE 1

A

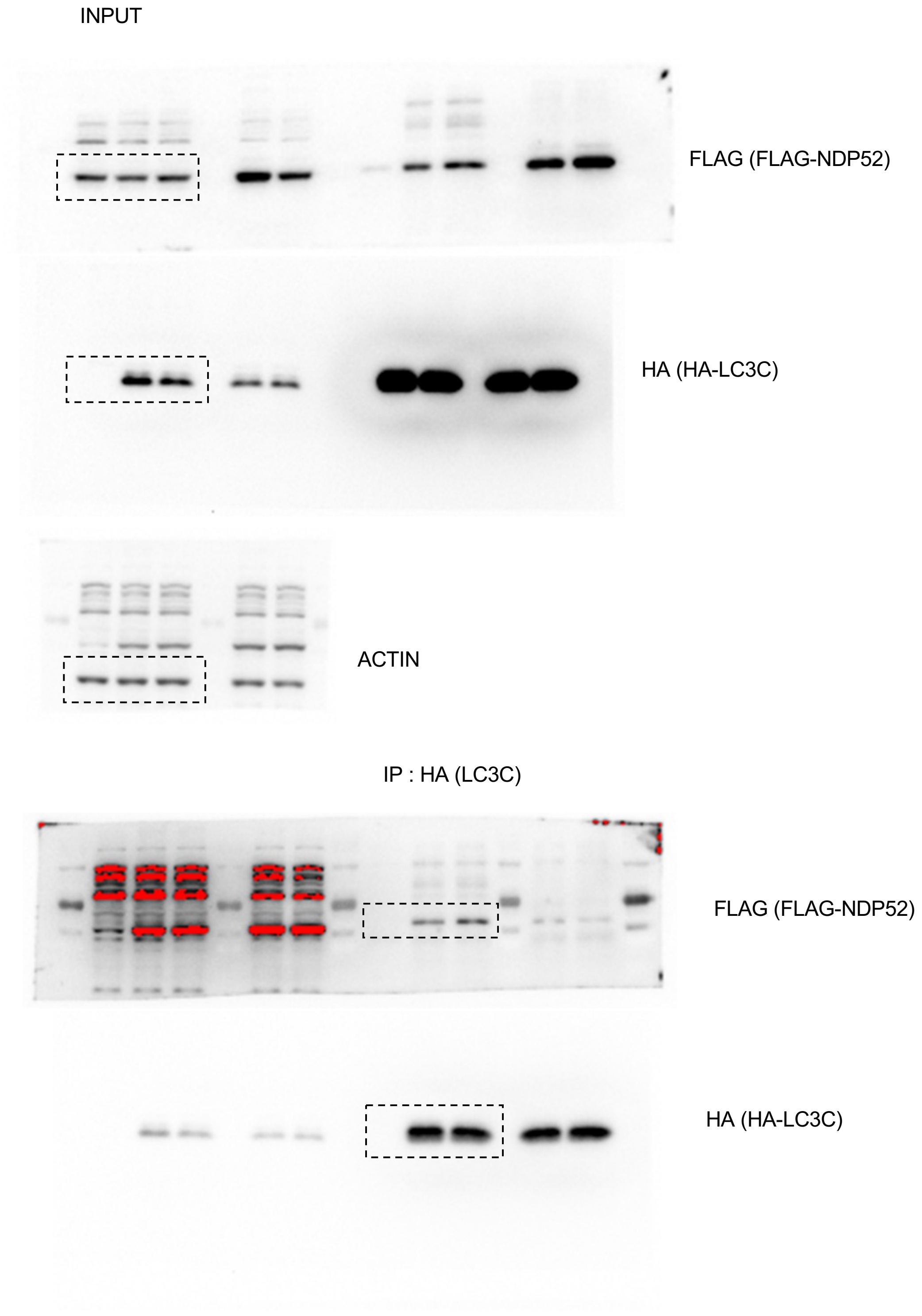
INPUT
FLAG (FLAG-NDP52)
HA (HA-LC3C)
ACTIN
IP : HA (LC3C)
FLAG (FLAG-NDP52)
HA (HA-LC3C)

FIGURE 1

B

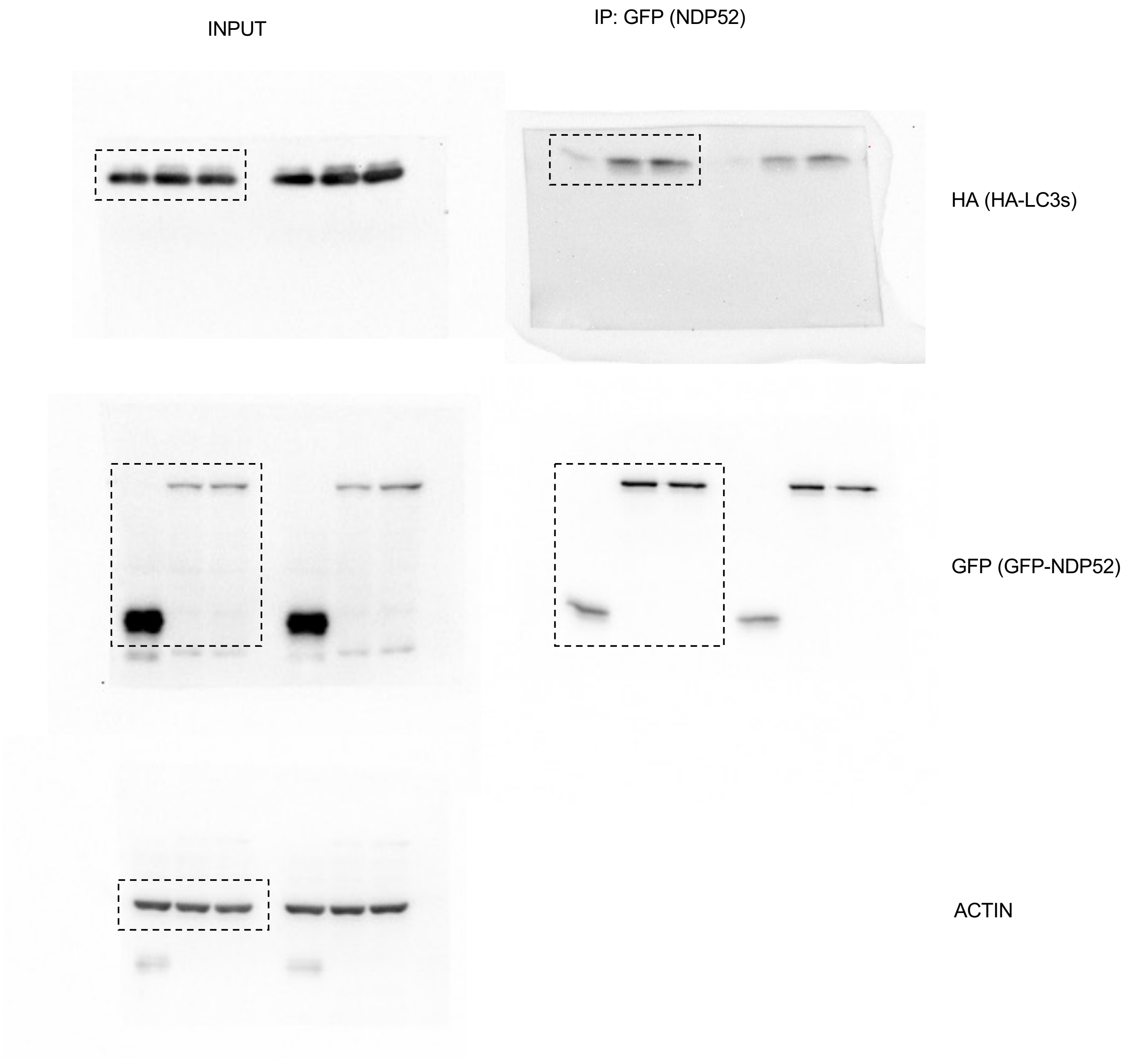

FIGURE 2

B

INPUT

IP: HA (LC3C)

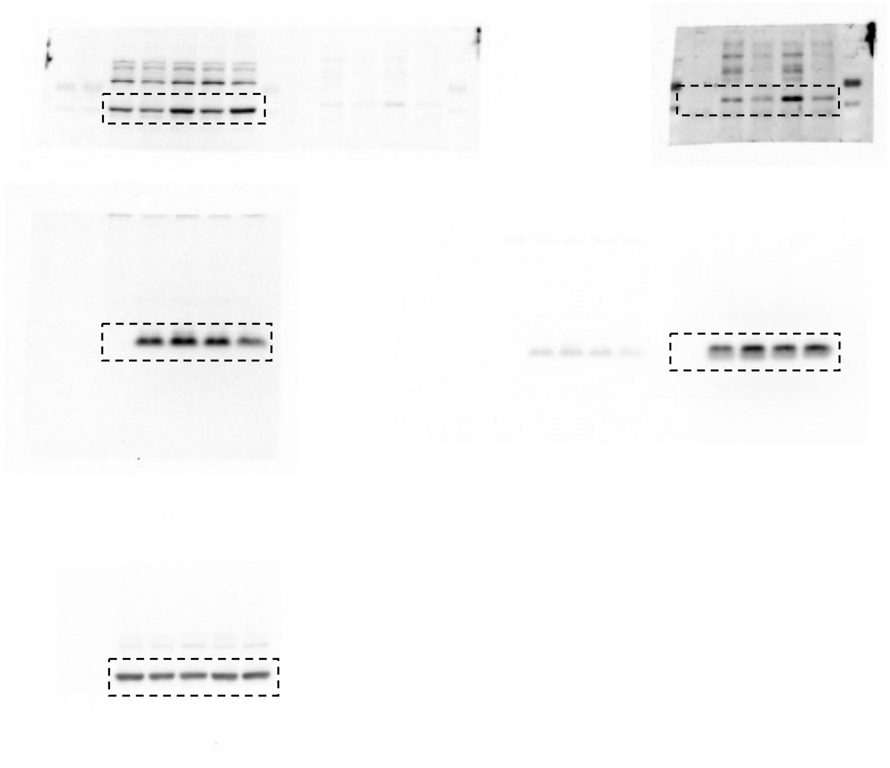

FLAG (FLAG-NDP52)

HA (HA-LC3C)

ACTIN

C

INPUT

IP: HA (LC3C)

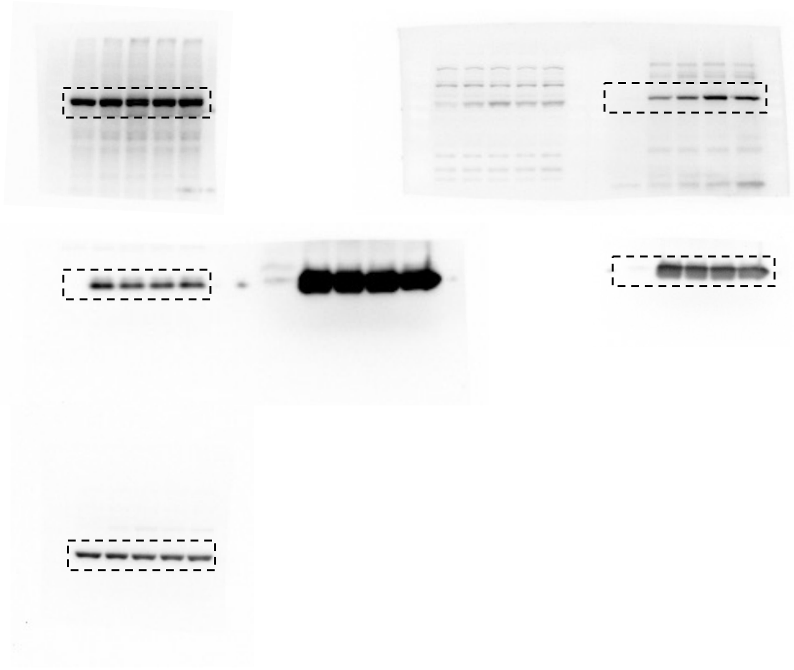

FLAG (FLAG-NDP52)

HA (HA-LC3C)

ACTIN

FIGURE 3

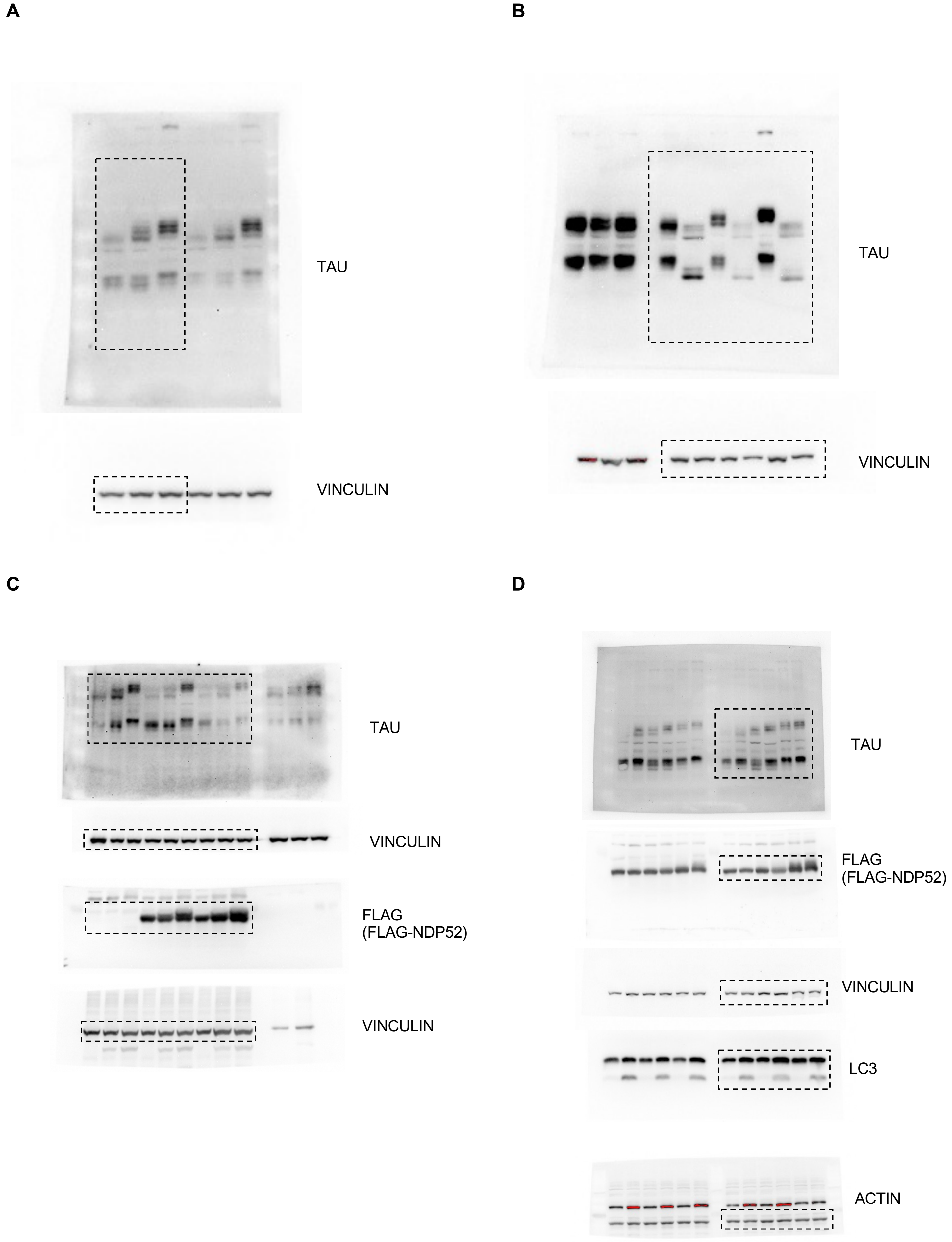
A
TAU
VINCULIN
B
TAU
VINCULIN
C
TAU
VINCULIN
FLAG
(FLAG-NDP52)
VINCULIN
D
TAU
FLAG
(FLAG-NDP52)
VINCULIN
LC3
ACTIN

FIGURE 4

A

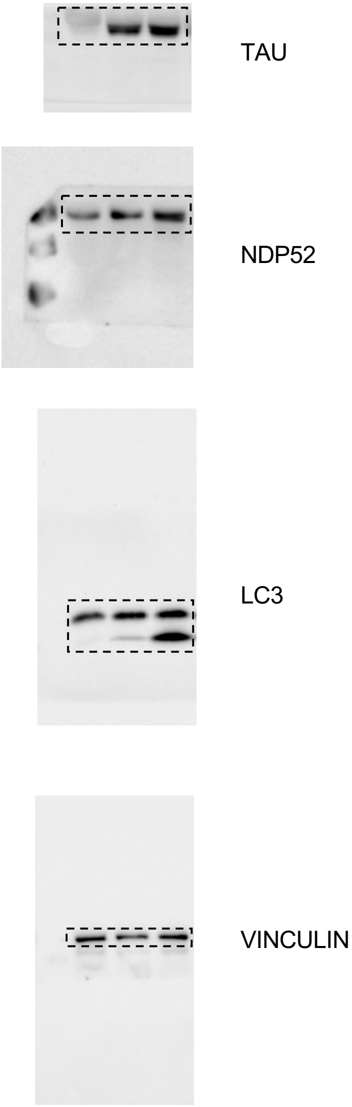

B

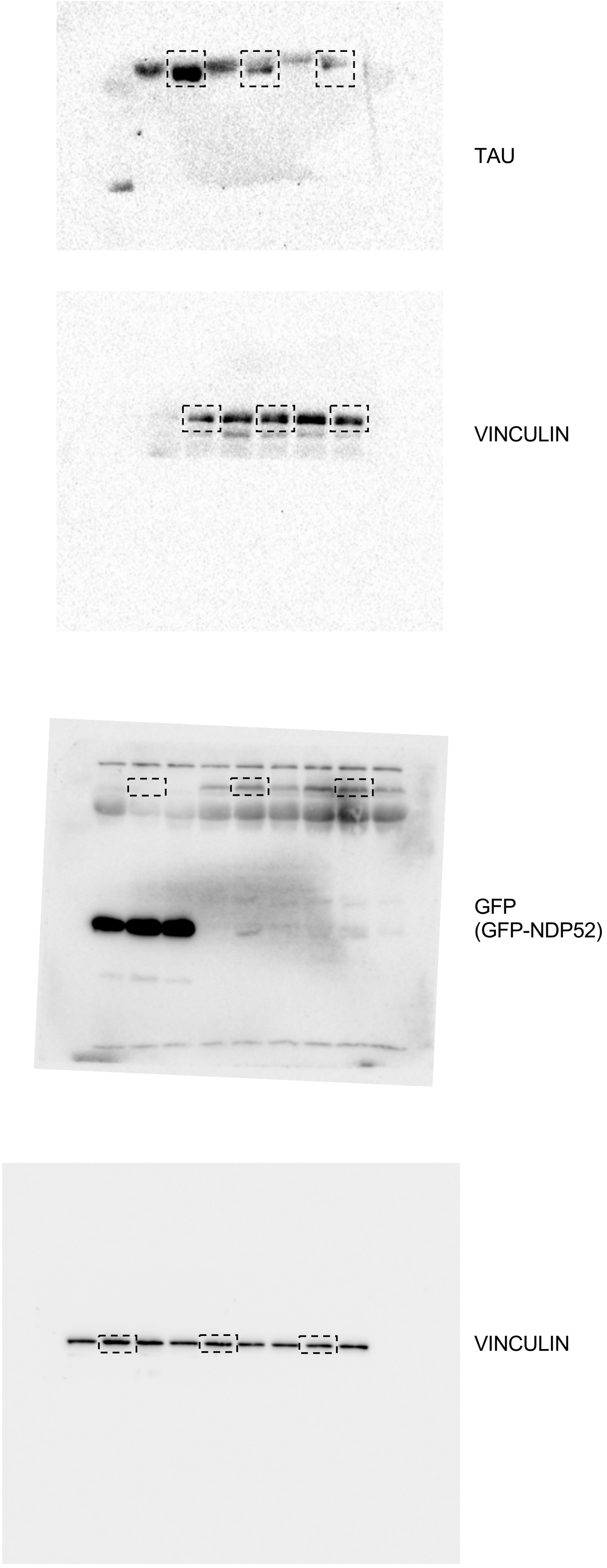

FIGURE 5

A

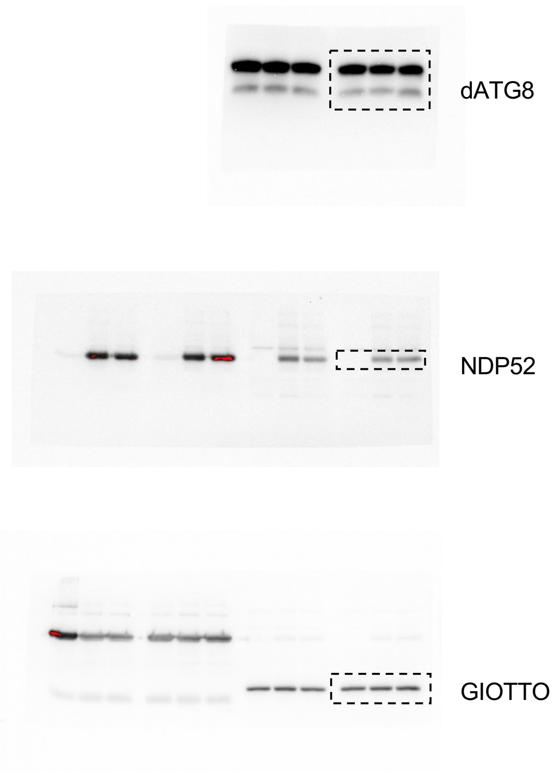

dATG8
NDP52
GIOTTO

E

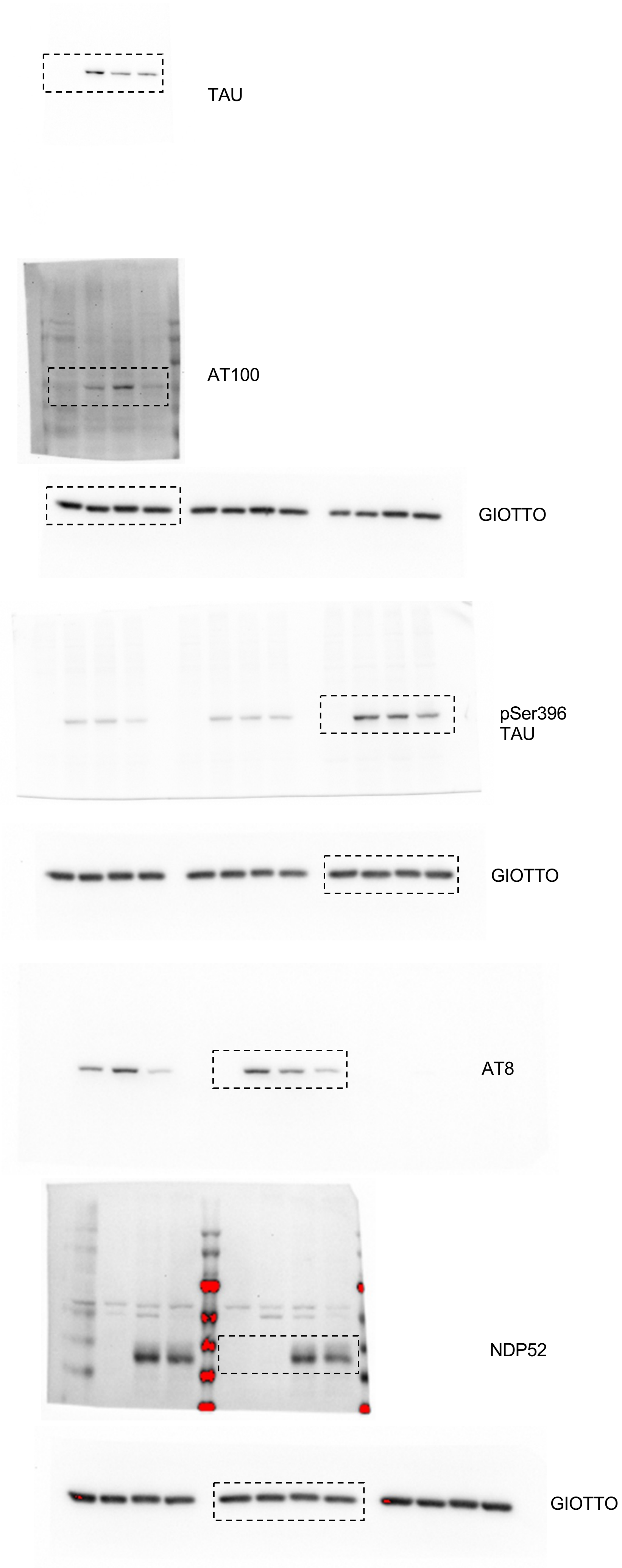

TAU
AT100
GIOTTO
pSer396
TAU
GIOTTO
AT8
NDP52
GIOTTO

Supplementary Figure 1

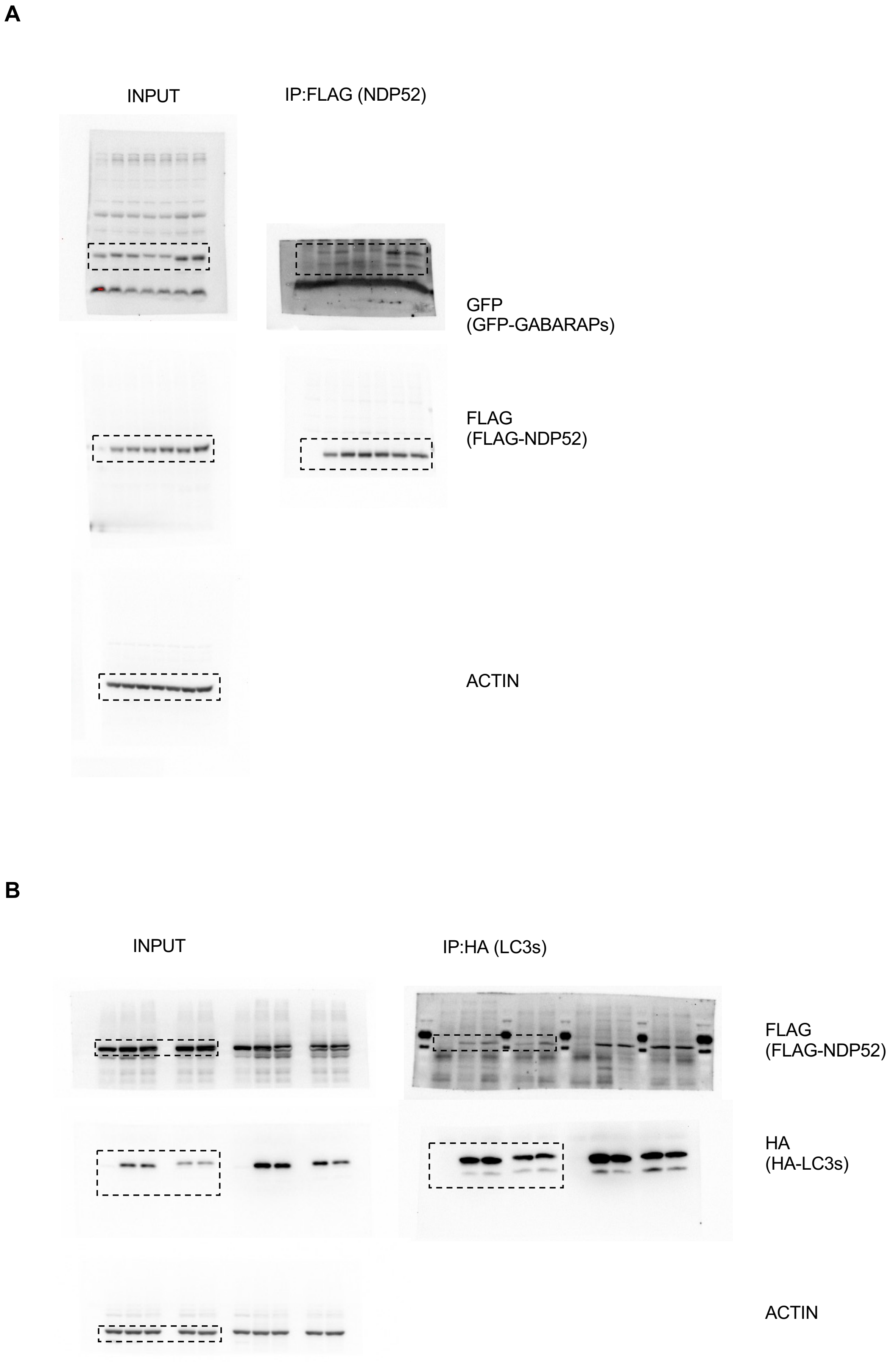

Supplementary Figure 2

A

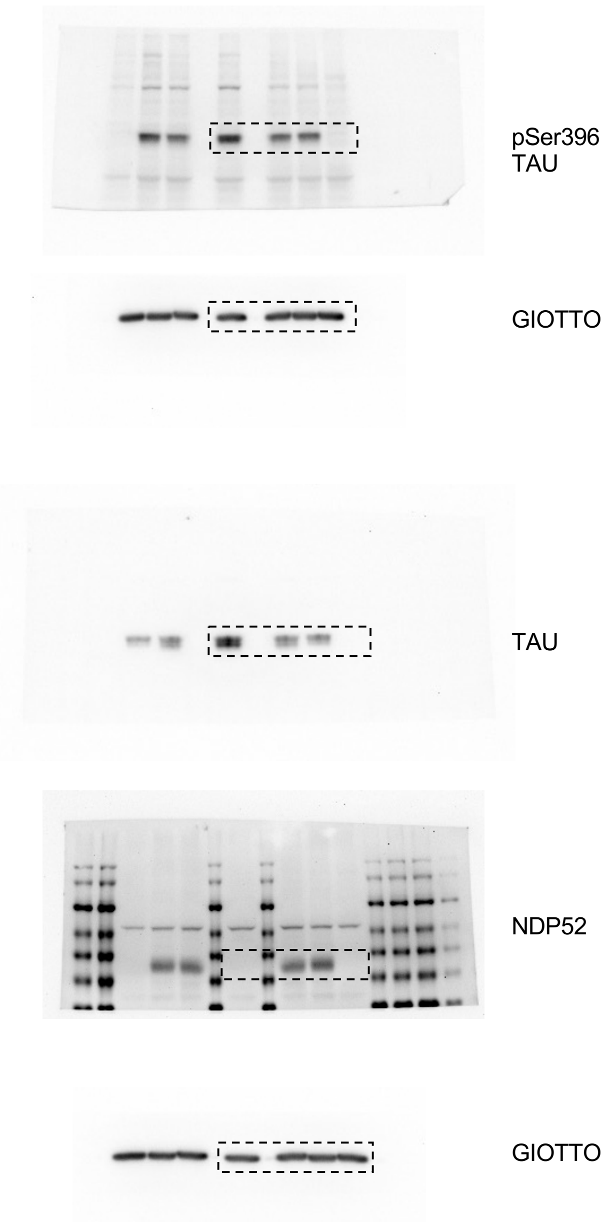

C

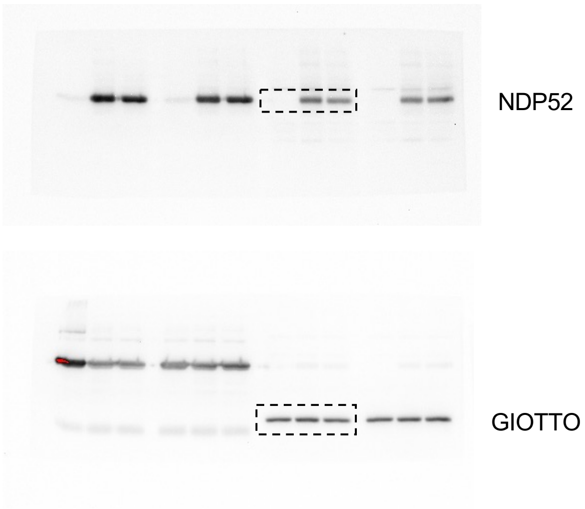

D

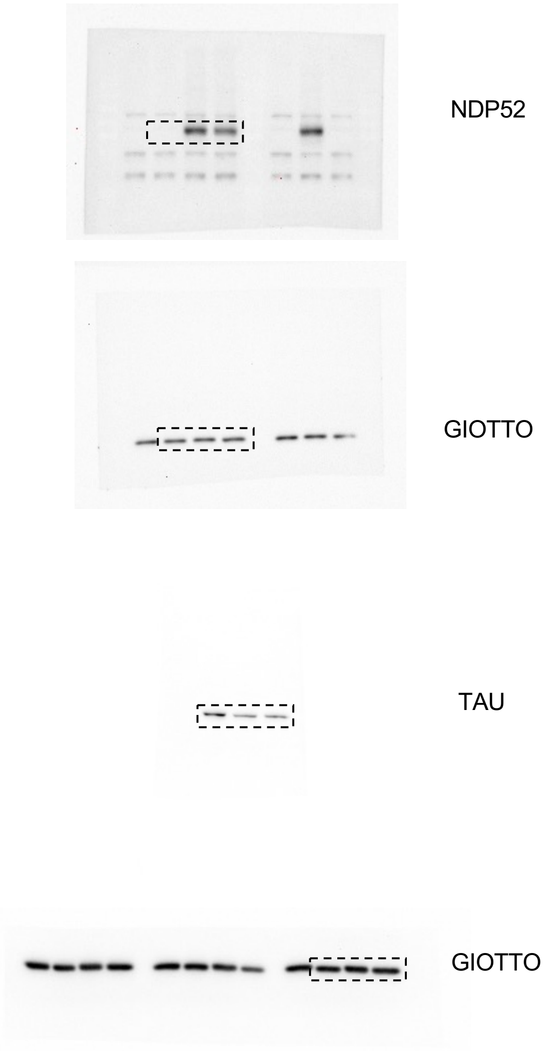

Supplement: Supplementary file 2 — Uncropped WB [file 41419_2025_7611_MOESM2_ESM.pdf]
